# Psychophysiological responses to a multimodal physiotherapy program in fighter pilots with flight-related neck pain: A pilot trial

**DOI:** 10.1371/journal.pone.0306708

**Published:** 2024-07-05

**Authors:** Carlos Fernández-Morales, Luis Espejo-Antúnez, María de los Ángeles Cardero-Durán, Deborah Falla, Juan Manuel Moreno-Vázquez, Manuel Albornoz-Cabello

**Affiliations:** 1 Department of Medical-Surgical Therapy, Faculty of Medicine and Health Sciences, University of Extremadura, Badajoz, Spain; 2 Centre of Precision Rehabilitation for Spinal Pain (CPR Spine), College of Life and Environmental Sciences, School of Sport, Exercise and Rehabilitation Sciences, University of Birmingham, Birmingham, United Kingdom; 3 Department of Physiotherapy, University of Seville, Seville, Spain; 4 Department of Physiology, Faculty of Medicine and Health Sciences, University of Extremadura, Badajoz, Spain; UFSCar: Universidade Federal de Sao Carlos, BRAZIL

## Abstract

**Background:**

The physical and cognitive demands of combat flying may influence the development and persistence of flight-related neck pain (FRNP). The aim of this pilot study was to analyse the effect of a multimodal physiotherapy program which combined supervised exercise with laser-guided feedback and interferential current therapy on psychophysiological variables in fighter pilots with FRNP.

**Methods:**

Thirty-one fighter pilots were randomly assigned to two groups (Intervention Group: n = 14; Control Group: n = 17). The intervention consisted of 8 treatment sessions (twice per week) delivered over 4 weeks. The following primary outcomes were assessed: perceived pain intensity (Numeric Pain Rating Scale–NPRS) and Heart Rate Variability (HRV; time-domain, frequency-domain and non-linear variables). A number of secondary outcomes were also assessed: myoelectric activity of the upper trapezius and sternocleidomastoid, pain catastrophizing (Pain Catastrophizing Scale–PCS) and kinesiophobia (TSK-11).

**Results:**

Statistically significant differences (p≤0.05) within and between groups were observed for all outcomes except for frequency domain and non-linear HRV variables. A significant time*group effect (one-way ANOVA) in favour of the intervention group was found for all variables (p<0.001). Effect sizes were large (d≥0.6).

**Conclusions:**

The use of a multimodal physiotherapy program consisting of supervised exercise with laser-guided feedback and interferential current appears to show clinical benefit in fighter pilots with FRNP.

**Trial registration:**

ClinicalTrials.gov: NCT05541848.

## Introduction

Combat flying exposes fighter pilots to highly stressful situations, which can affect their physical and cognitive performance [1, 2]. Exposure to high gravitational accelerations (G-forces) and the operation of heavy technological equipment may influence the development of flight-related neck pain (FRNP) [3, 4].

FRNP impacts a pilot’s ability to perform critical tasks and is associated with a physiological stress response [4–7]. As a result, NATO recommends monitoring physiological parameters such as Heart Rate Variability (HRV) to assess symptoms and clinical signs in fighter pilots with FRNP [4]. Nevertheless, to date, these measures have only been used to assess flight performance [1, 2].

In the workplace, it has been observed that workers with musculoskeletal pain may show a decrease in HRV [8], with a marked reduction in parasympathetic activity [9]. An autonomic dysregulation has also been related to alterations in the electrical activity of muscles [10]. The role of psychological responses as modulators of the autonomic nervous system of people with chronic neck pain has also been investigated [11]. Parr et al. [12] indicated that pain catastrophism and fear of movement (i.e., kinesiophobia) influence the persistence of pain in this population, with HRV being an indicator that relates to certain psychological phenomena associated with the experience of pain [8].

Combined therapeutic interventions such as exercise [6], manual therapy [13] or interferential current therapy (ICT) [14–16] have been shown to be most effective in musculoskeletal back disorders [17]. A novel procedure within this approach is electrostimulation therapy known as electromassage, which applies ICT together with manual therapy simultaneously [18]. This modality has shown clinical benefits due to its ability to penetrate deeper into tissues and its greater tolerance to low-frequency currents [17]. In addition, ICT has been shown to induce psychophysiological effects such as changes in HRV when these techniques are combined within a multimodal physiotherapy program [14]. With respect to exercise, a recent systematic review found that cervical motor control exercises are more effective in reducing pain in fighter pilots with FRNP than exercise protocols which only involve strength or endurance [19]. Moreover, the combination of these exercises guided with an external locus (e.g., a laser pointer) further improves the outcome [20, 21].

Recent studies analyzing work-related musculoskeletal disorders have stated that the lack of studies in this context is due to the difficulty in accessing the population, which makes sampling difficult [22]. Despite this, and to the best of the authors’ knowledge, there are no studies analysing the psychophysiological responses derived from a multimodal physiotherapy in fighter pilots with FRNP. The hypothesis is that a multimodal physical therapy program will produce significant improvements in psychophysiological parameters compared to a control group receiving no intervention. Therefore, the aim of this pilot study was to investigate the effects of a multimodal physiotherapy intervention on psychophysiological parameters in fighter pilots with FRNP.

## Materials and methods

### Study design

A randomized controlled single-blind pilot trial was conducted with concealed allocation, intention-to-treat analysis and with a blinded assessor and statistician. The study was carried out in compliance with recommendations of CONSORT [23]. The present study was conducted following the Declaration of Helsinki and was approved by the Ethical Research Committee of the University of Extremadura (approval number 54/2020). It was also registered prospectively at ClinicalTrials.gov (NCT05541848). All participants provided written informed consent.

### Participants

**Fig 1:**
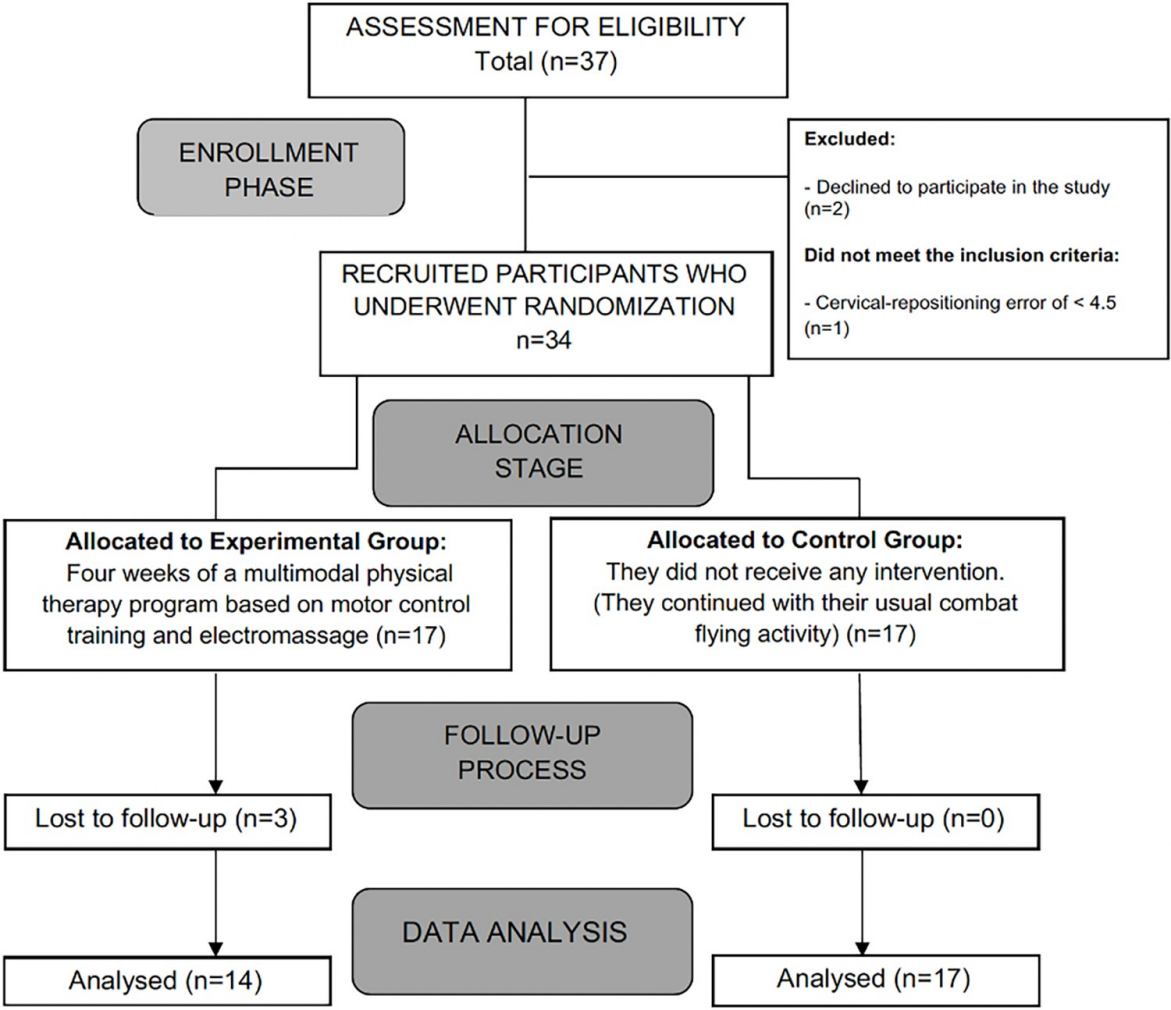
Flow-chart diagram of the progress of patients through the study phases.

Via convenience sampling, recruitment took place from September 2022 to December 2022 at Talavera´s Air Base in Southern Spain. An initial, potentially eligible sample of 37 F-5 fighter pilots were recruited. The inclusion criteria were: (i) fighter pilots (male and female) who, at the time of the assessment, were an instructor or student attached to the 23th Wing of Talavera Air Base, Spanish Air Force (SAF), Badajoz; (ii) fighter pilots diagnosed with FRNP, defined by NATO [4] as significant neck pain that occurs during or within 48 hours after the flight; it does not refer to pain that is attributed to other activities or causes; (iii) a minimum perceived pain of 3/10 on the Numeric Pain Rating Scale (NPRS) in an early-morning assessment within 48 hours of the last flight. The exclusion criteria were: (i) Personal Psychological Apprehension Scale (PPAS) score higher than 37.5 [24]; (ii) any contraindication for electrical stimulation; (iii) having received physiotherapy or any other routine medical care six weeks prior to data collection; (iv) any regular use of drugs within the prior two weeks before participating in this study that affect the autonomic nervous system or pain perception, including opioids, antidepressants, benzodiazepines, anti-inflammatory drugs, and beta-blockers; (v) being involved in ongoing medical–legal conflicts. The total sample consisted of 31 fighter pilots. Fig 1 provides a flow diagram of participant recruitment and flow through the study.

### Randomization

An external website (http://www.randomization.com) (accessed on 27 August 2022) was used to complete the group allocation. Participants were randomly (using block randomization, 1:1) allocated into 1 of the 2 groups: the intervention group (IG) and the control group (CG). The randomization was performed by an external assistant who was not involved in recruitment.

### Blinding

Two researchers carried out the study; a blinded researcher collected the measurements at baseline and immediately after the treatment whereas another researcher carried out the intervention. Both were physiotherapists with more than 15 years of experience.

The allocation process was conducted in a protected area to ensure that both the examiner and the intervention provider remained blind.

### Intervention

**Fig 2:**
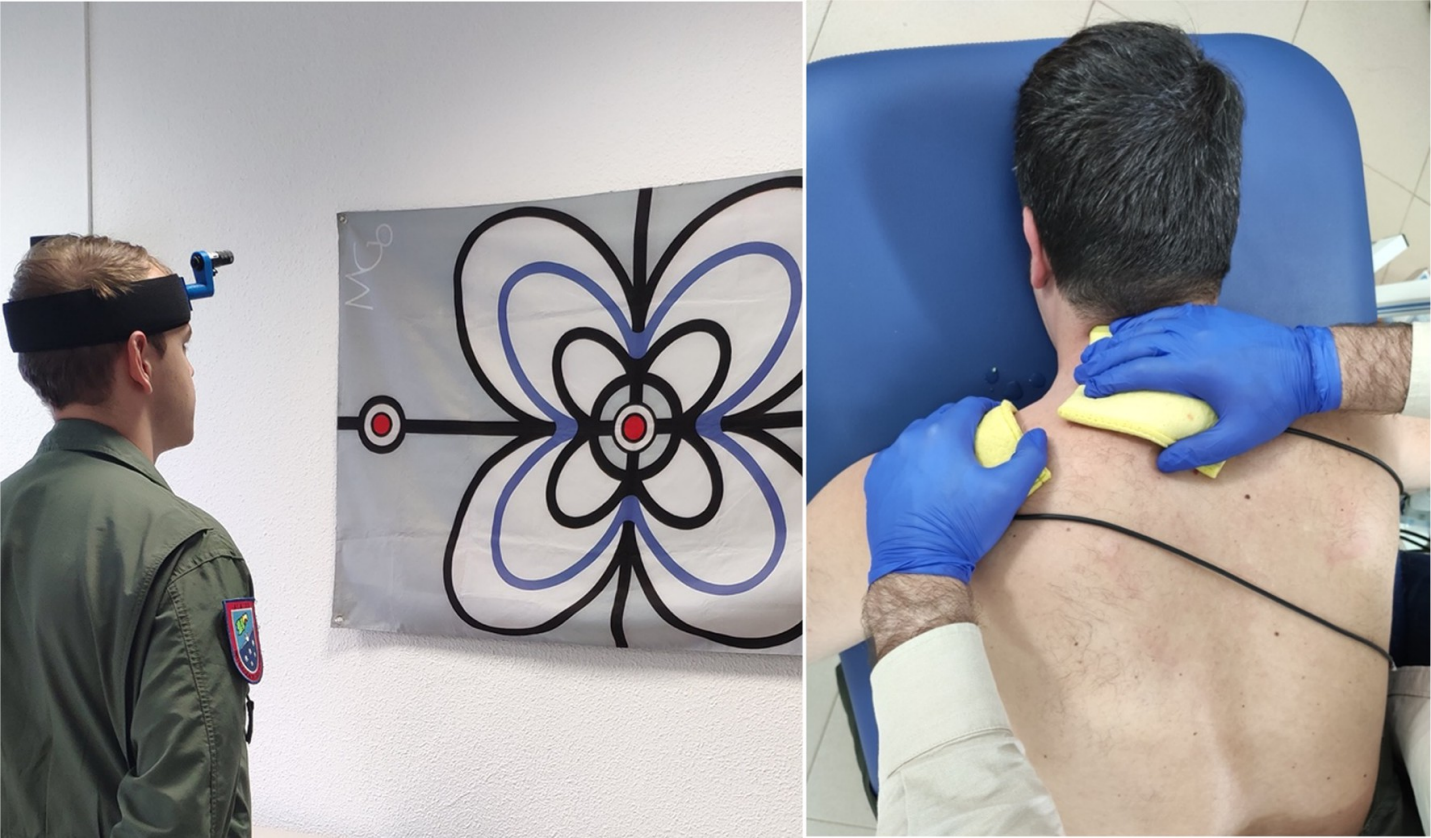
Illustration of ELGF and ICE interventions.

The participants in the IG (n = 14) followed a program of supervised neck-specific exercise with laser-guided feedback (ELGF) (S1 Appendix). Afterwards, they received an intervention based on manual therapy combined with current interferential therapy called electro-massage [25] (S2 Appendix). They completed a total of 8 sessions over 4 weeks (twice per week). The multimodal treatment program was carried out within 48 hours after their last flight and was carried out at Talavera´s Air Base (Spain) (Fig 2). The participants of the control group (n = 17) did not receive any intervention and continued with their normal combat and exercise flying activities. They were asked not to take medication or to seek alternative treatments. The interventions were led by 2 physical therapists. An adherence rate to the intervention of 75% (6 sessions) was established as a minimum for participants to be included in the final analysis.

#### Neck-specific supervised exercises with laser-guided feedback (ELGF)

ELGF is defined as a type of therapeutic exercise characterised by requiring the individual to pay attention to head/neck movement. This type of exercise (also called internal focus/locus of movement control exercise) play an important role in motor skill learning [20]. This has been attributed a to the facilitation of automatic control and retention of movement patterns in subjects with spinal pain [21, 26].

The "Motion Guidance Clinician Kit" (Motion Guidance LLC, Denver, CO, USA.) was used, consisting of a panel and laser guide positioned on the forehead for precise execution of neck movements. The laser was positioned by means of an elastic band at the pilot’s forehead. The panel was placed 1.5 metres away from the wall. The program consisted of 4 exercises, which progressed in difficulty according to the tolerance achieved over the course of the sessions: a) Maintaining the head position (cervical stabilisation); b) Cervical flexion-extension; c) Right-left rotations; d) Right-left lateral-flexions. Each exercise consisted of 4 series of 8 repetitions, except the first one. The head position is maintained by pointing the laser at the centre of the panel for 30 seconds (4 series) (S1 Appendix). Between each series there was 10 seconds of rest. The subjects began seated on a stool and then moved to standing from the 3^rd^ session onwards. From the 5^th^ session onwards, the distance between the signals to be reached with the laser was increased, with the aim of increasing the range of cervical movement in the 3 planes of space. The interventions were carried out in compliance with the recommendations of the CERT [27] and TIDieR [28] statements.

#### Interferential current electro-massage

Electro-massage is a novel procedure that simultaneously combines interferential current with manual therapy [25], involving a 15-minute treatment performed in the cervical region. We used a current bipolar mode, using a carrier frequency of 4000 Hz at constant voltage and an amplitude-modulated frequency of 100 Hz (Sonopuls 692®; Enraf-Nonius BV, Rotterdam, The Netherlands). The therapist applies manual soft tissue therapy while administering ICT through moistened sponges, focusing on the neck, shoulder, and scapular regions. The protocol includes various techniques such as superficial strokes, deep sliding movements, kneading of the upper trapezius, and gentle stretching of cervical muscles [25]. During the manual therapy, the sliding of the electrodes was accompanied by the necessary pressure according to each phase of the electro-massage sequence (S2 Appendix).

### Outcome measures

Participants were evaluated in separate rooms within their workplace, with the rooms at the same temperature, in order to maintain the environmental conditions consistent between participants. All measures were conducted within 48 hours after the flight, in both the baseline and final assessment, in the same order and under the same conditions for all participants.

HRV was recorded in both groups with participants in a prone position and this was conducted in the early morning after fasting overnight [29]. The recording was performed weekly: the first day of the intervention week for the IG after the end of the treatment; and the first day of the week for the CG at the same time and in the same position, to serve as a control for this group. Finally, the mean of all measurements taken in both groups was calculated. The rater, who was blinded to group allocation, collected the baseline clinical data. Demographic, anthropometric, and clinical data were collected using a self-assessment questionnaire created for this study.

### Primary outcomes

#### Neck pain intensity—Numeric Pain Rating Scale

The Numeric Pain Rating Scale (NPRS) is a 11-point numeric rating scale, where 0 denotes “no pain” and 10 denotes “the maximum pain imaginable”. The minimum clinically important difference (MCID) for this tool has been established at 1.5 points and the minimum detectable change (MDC) at 2.6 points, in individuals with neck pain. The NPRS is a valid scale with moderate test-retest reliability in this population (Intraclass Coefficient Correlation (ICC): 0.76, 95% CI 0.58 to 0.93) [30, 31].

#### Heart rate variability

HRV is a non-invasive marker which reflects changes in the autonomic response associated with stress [15]. Interbeat time interval (R-R) variation was used to determine the autonomic modulation using Firstbeat Bodyguard equipment (Firstbeat Technologies, Jyväskylä, Finland). This device was used to record HRV data for 15 min (at rest and after the intervention). Recordings were exported from the devices to the computer via Firstbeat Uploader Software (Firstbeat Technologies) and analysed using Kubios Software (University of Eastern Finland, Kuopio, Finland). To calculate the autonomous balance, the HRV method based on the Poincaré plot was used [29, 32]. This software has proven to be valid and capable of recording non-linear trends that are frequently present on R-R intervals of record [15]. Recommendations of the Task Force of the European Society of Cardiology and the North American Society of Pacing and Electrophysiology [33], as well as instructions derived from previous studies that used the heart rate monitor Firstbeat Bodyguard®, were followed [29].

Mean heart rate and time domain parameters were measured: percentage of consecutive RR intervals that differ by more than 50 ms from each other (pNN50) and root mean square of successive differences (rMSSD); frequency-domain parameters: low-frequency power (LF), hight-frequency power (HF) and low/high-frequency ratio (LF/HF); diameters of Poincaré plot, the short-term variability’s sensitivity of HRV non-linear specter (SD1); and the long-term variability of HRV non-linear specter (SD2). rMSSD and SD1 are considered indicators of parasympathetic activity [14, 15]. The physiological meaning of SD2 is not entirely clear, but it is thought to reflect the long-term changes in RRIs, and it is considered an inverse indicator of sympathetic activity [14, 15]. Naranjo-Orellana et al. [32] described two new indexes to simplify the physiological interpretation of Poincaré plot: the stress score (SS) and sympathetic-parasympathetic ratio (S/PS). The SS is expressed as the inverse of SD2 diameter multiplied by 1000, and it is directly proportional to sympathetic activity at the sinus node. The S/PS ratio is expressed as the quotient of SS and SD1, and it is considered to reflect autonomic balance, that is, the relationship between sympathetic and parasympathetic activity.

### Secondary outcomes

#### Myoelectric activity

**Fig 3:**
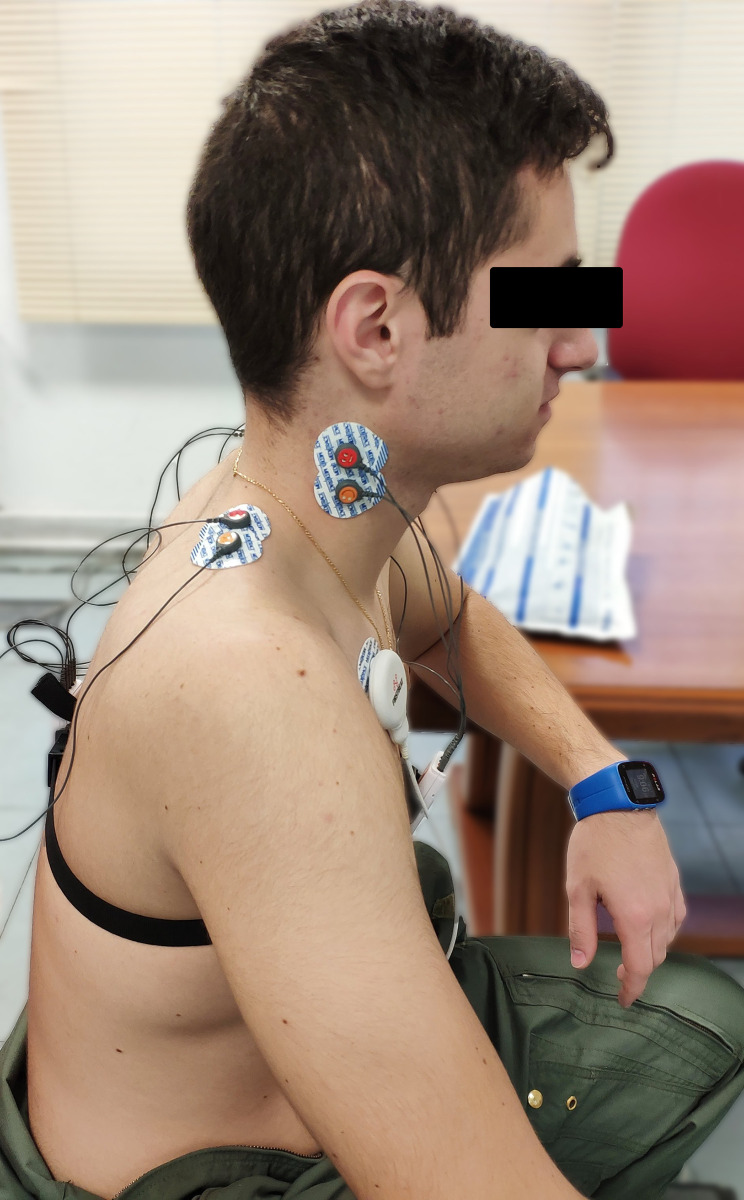
Detail of the placement of the EMG electrodes.

Myoelectric activity was measured using surface electromyography (sEMG) following NATO expert panel recommendations [4]. The sEMG recordings were acquired using a portable EMG system (mDurance® system, mDurance Solutions SL, Granada, Spain), which has been validated for measuring muscle activity during dynamic contractions [34]. Disposable electrodes (35x42 mm, Ag/AgCl, Blue Sensor N-00-S, Medicotest A/S, Olstykke, Denmark) were placed pairwise on the upper trapezius and sternocleidomastoid muscles according to established guidelines [35, 36] following skin preparation. A reference electrode was placed over the acromion (Fig 3).

The raw data were processed and filtered using a fourth-order Butterworth bandpass filter with a 20–450 Hz cut-off frequency. The maximum voluntary contraction (MVC) value was calculated using the average of the three maximum peaks of the root mean square (RMS) during MVC contractions [34].

The pilots sat in the cockpit of the flight simulator, reproducing the position and motion during combat flight. Familiarization tests consisted of the participant performing shoulder elevation and cervical retraction, pushing against the resistance offered by the seat. Participants were then placed in a neutral cranio-cervical position, and were instructed to perform the same contractions and push with increasing force up to a MVC force and hold for this 5 s. Three trials were done with 1 min rest in between each repetition [37]. The variable recorded for muscle activity was the mean RMS measured during MVC (μV).

#### Catastrophizing

The Spanish version of the Pain Catastrophizing Scale (PCS) is a self-administered scale (Likert scale) of 13 items and one of the most used and reliable to assess pain catastrophizing in military personnel [38]. Participants were asked to refer to their past painful experiences and indicate the degree to which they experienced each of the 13 thoughts or feelings; the score ranges from 0 (never) to 4 (always).

#### Kinesiophobia

The Spanish version of the Tampa Kinesiophobia Scale (TSK-11) was used [39]. This questionnaire contains 11 items to assess the patient’s fear of moving and re-injury. Each item is associated with a 4-point Likert scale (1 = “strongly disagree”, 4 = “strongly agree”). Scores range from 11 to 44 points; higher scores correspond with a greater fear of pain, movement and injury. The Spanish version of the TSK-11 has shown good reliability and validity (Cronbach alpha: 0.79) [39].

### Sample size

Sample size calculation for detecting changes in the primary outcome (NPRS scale) was performed using G*power 3.1 software. The calculations were based on the minimal clinically important difference (MCID) of 2.5 points in the NPRS scale (estimated for a variance of 10 points in patients with neck pain) [31], assuming a standard deviation (SD) of 2.5 points. Considering an effect size (F-test) of 0.27 for ANOVA: repeated measures, within-between interaction groups differences, an alpha level of .05, and a power of 80%, a total sample size of 30 participants was estimated. To account for potential dropouts, the sample was inflated by 10%, resulting in a final target sample size of 34. This calculation indicated that a sample size of 17 participants per group was required to achieve a 95% confidence interval with 80% power.

### Statistical analysis

An intention-to-treat analysis was conducted. Descriptive analysis was conducted for each variable. Normality of the variables was assessed using the Shapiro-Wilk test, which indicated a normal distribution for all variables, allowing for the use of parametric tests. Mean ± SD was used to report the data. To compare the demographic and clinical variables between groups at baseline, the chi-square test was employed for categorical data, while the independent-samples t-test was used for quantitative data. Differences in measurements were detected by analysis of variance of repeated measures (ANOVA) 2x2, mixed analysis of variance (ANOVA) to evaluate group x time interactions*, including the effect of time (baseline, four weeks after treatment) as intra-subjects’ factor and group effects (Intervention Group vs Control Group) as inter-subjects’ factors. Eta square (η^2^) was used to calculate the effect size (small when 0.01≤ η^2^≤ 0.06; medium when 0.06≤ η^2^> 0.14; large when η^2^> 0.14). Statistical significance was determined at p<0.05. Additionally, Cohen’s d coefficient was calculated to determine the effect size, with values above 0.8 considered high, 0.5 considered moderate, and less than 0.2 considered low [40]. Statistical significance was set at p < 0.05. Data analysis was carried out using SPSS version 26.0 (SPSS Inc., Chicago, IL, USA).

## Results

Table 1 lists the baseline clinical and demographic features of the participants. There were no significant differences between groups at baseline for any of the variables (all, p > 0.05). Three participants dropped out of the IG due to work-related demands. No adverse events were recorded.

**Table 1:** Baseline characteristics of participants (mean ± standard deviation).

	Total sample (n = 31)	Intervention Group (n = 14)	Control Group (n = 17)
**Age (years)**	25 ± 6.4	24 ± 5.5	26 ± 7.1
**Height (cm)**	178 ± 6.5	179 ± 5.8	178 ± 7.1
**Weight (kg)**	76.2 ± 8.4	77.7 ± 6.8	75.0 ± 9.6
**BMI (kg/m** ^**2**^ **)**	23.8 ± 1.9	24.0 ± 1.4	23.6 ± 2.2
**Number of flight hours / week**	4.4 ± 1.5	3.8 ± 1.2	4.8 ± 1.6
**Number of hours of physical activity / week**	3.6 ± 1.7	3.9 ± 1.4	3.3 ± 1.9
**NPRS (0–10)**	5.5 ± 1.7	6.1 ± 1.8	5.1 ± 1.5
**PCS (0–62)**	17 ± 6.1	18 ± 6.5	17 ± 6.0
**TSK-11 (1–68)**	36 ± 2.6	36 ± 2.8	35 ± 2.5
**RMS UT Right (μV)**	152.1 ± 49.5	152.4 ± 21.4	146.3 ± 29.2
**RMS UT Left (μV)**	164.9 ± 48.1	160.9 ± 56.2	168.2 ± 41.9
**RMS SCM Right (μV)**	19.5 ± 11.6	18.6 ± 8.7	20.3 ± 13.8
**RMS SCM Left (μV)**	21.3 ± 13.4	20.2 ± 10.6	22.3 ± 15.5
**Mean HR (bpm)**	74.8 ± 13.7	74.3 ± 15.3	75.1 ± 12.7
**pNN50 (%)**	13.5 ± 16.5	11.8 ± 12.4	14.9 ± 19.6
**rMSSD (ms)**	30.1 ± 10.6	29.5 ± 12.9	30.6 ± 8.5
**LF (ms** ^**2**^ **)**	1484.3 ± 789.3	1519.4 ± 757.7	1455.4 ± 836.4
**HF (ms** ^**2**^ **)**	294.1 ± 139.2	259.5 ± 106.5	322.6 ± 158.7
**LF/HF ratio**	5.4 ± 3.1	6.4 ± 3.9	4.6 ± 1.9
**SD1 (ms)**	21.4 ± 7.1	21.6 ± 8.6	21.2 ± 5.8
**SD2 (ms)**	61.9 ± 27.9	63.5 ± 37.1	60.6 ± 18.4
**SS (ms)**	18.6 ± 6.9	19.6 ± 9.2	17.7 ± 4.6
**S/PS ratio**	1.1 ± 0.8	1.3 ± 1.1	0.9 ± 0.4

Data are reported as mean ± SD. BMI: Body Mass Index; NPRS: Numeric Pain Rating Scale; PCS: Pain Catastrophizing Scale; TSK-11: Tampa Scale of Kinesiophobia; RMS: Root mean square; UT: Upper trapezius; Mean HR = Average heart rate, beats per minute (bpm); SCM = Sternocleidomastoid; SD1 = transverse axis of Poincaré plot millisecond (ms); SD2 = longitudinal axis of Poincaré plot, SS = stress score (inverse of diameter SD2 x 1000); S/PS ratio = quotient of SS and SD1; Min HR: Minimum heart rate variability; Max HR: Maximum heart rate variability, beats per minute (bpm).

Table 2 shows the baseline and post-intervention scores and the mean differences between and within groups for all the variables. Compared to baseline values, the IG showed significant changes (p<0.001) and moderate effect sizes (*d*≥0.6) for PCS, RMS, pNN50 and rMSSD, and large effect sizes (*d*≥0.8) for NPRS, TSK-11, RMS measured during MVC and SD1.

**Table 2:** Baseline, post-intervention and mean score changes.

	Groups	Baseline	Post-Intervention	Pre-/post-differences	Between-groups mean changes Post-Intervention
**NPRS (0–10)**	Intervention	6.1 ± 1.8	1.6 ± 1.6	4.5 [3.3–5.5]** (0.8)	2.5 [1.5–3.5]^††^ (0.7)
	Control	5.1 ± 1.5	4.1 ± 1.0	1.0 [0.5–1.4]* (0.3)	
**PCS (0–62)**	Intervention	18 ± 6.5	8 ± 5.0	10 [6.3–12.2]** (0.7)	8 [3.3–10.9]^†^ (0.6)
	Control	17 ± 6.0	16 ± 5.3	1 [0.8–3.2]* (0.1)	
**TSK-11 (1–68)**	Intervention	36 ± 2.8	23 ± 6.2	13 [9.8–15.8]** (0.8)	8 [4.3–12.5]^††^ (0.6)
	Control	35 ± 2.5	31 ± 4.9	4 [1.3–6.1]* (0.4)	
**RMS UT Right (μV)**	Intervention	152.4 ± 21.4	194.8 ± 32.5	42.4 [23.7–61.0]** (0.6)	43 [19.6–66.5]^†^ (0.6)
	Control	146.3 ± 29.2	151.8 ± 30.7	5.5 [-27-6-16.8]	
**RMS UT Left (μV)**	Intervention	160.9 ± 56.2	204.7 ± 47.3	43.8 [17.7–69.8]* (0.4)	60.1 [30.3–89.9]^††^ (0.6)
	Control	168.2 ± 41.9	144.6 ± 33.6	23.6 [3.1–44.1]* (0.3)	
**RMS SCM Right (μV)**	Intervention	18.6 ± 8.7	42.3 ± 23.6	23.7 [11.5–35.7]* (0.6)	28 [15.0–40.1]^††^ (0.6)
	Control	20.3 ± 13.8	14.3 ± 10.0	6 [-1.9–13.8]	
**RMS SCM Left (μV)**	Intervention	20.2 ± 10.6	34.4 ± 18.4	14.2 [3.4–24.8]* (0.4)	15 [3.6–26.6]^†^ (0.4)
	Control	22.3 ± 15.5	19.2 ± 12.6	3.1 [-7.6–13.9]	
**Mean HR (bpm)**	Intervention	74.3 ± 15.3	62.3 ± 8.1	12.0 [3.1–20.7]* (0.4)	13 [5.6–19.7]^†^ (0.6)
	Control	75.1 ± 12.7	75.0 ± 10.5	0.1 [-6.5–6.6]	
**pNN50 (%)**	Intervention	11.8 ± 12.4	43.6 ± 20.3	31.8 [20.5–43.1]** (0.7)	28.7 [18.0–39.4]^††^ (0.7)
	Control	14.9 ± 19.6	14.8 ± 6.5	0.1 [-9.4–9.5]	
**rMSSD (ms)**	Intervention	29.5 ± 12.9	60.6 ± 19.2	31.1 [21.4–40.7]** (0.7)	25.1 [1.4–36.6]^††^ (0.7)
	Control	30.6 ± 8.5	35.5 ± 7.8	4.9 [0.2–10.1]	
**LF (ms** ^**2**^ **)**	Intervention	1519.4 ± 757.7	1601.0 ± 1342.8	81.5 [-831.1–668]	234.7 [-505.6–975.2]
	Control	1455.4 ± 836.4	1835.7 ± 598.7	380.3 [105.7–866.3]	
**HF (ms** ^**2**^ **)**	Intervention	259.5 ± 106.5	418.4± 215.3	158.9 [57.3–260.5]* (0.4)	62.3 [-100.3–224.9]
	Control	322.6 ± 158.7	476.4 ± 224.3	153.8 [56.9–250.5]* (0.3)	
**LF/HF ratio**	Intervention	6.4 ± 3.9	4.9 ± 4.3	1.5 [-0.9–4.1]	0.3 [-2.8–2.2]
	Control	4.6 ± 1.9	4.6 ± 2.3	0.01 [-1.4–1.4]	
**SD1 (ms)**	Intervention	21.6 ± 8.6	48.8 ± 14.6	27.2 [18.2–36.0]** (0.8)	22.6 [13.9–31.2]^††^ (0.7)
	Control	21.2 ± 5.8	26.2 ± 4.2	5.0 [1.0–9.0]* (0.4)	
**SD2 (ms)**	Intervention	63.5 ± 37.1	81.0 ± 34.8	17.5 [8.9–43.9]	12.7 [7.6–32.9]
	Control	60.6 ± 18.4	68.3 ± 19.6	7.7 [6.4–21.8]	
**SS (ms)**	Intervention	19.6 ± 9.2	14.9 ± 7.0	4.7 [-0.5–9.9]	0.7 [-3.7–5.1]
	Control	17.7 ± 4.6	15.6 ± 3.9	2.1 [0.8–5.1]	
**S/PS ratio**	Intervention	1.3 ± 1.1	0.4 ± 0.4	0.9 [0.3–1.4]* (0.5)	0.2 [-0.05–0.4]
	Control	0.9 ± 0.4	0.6 ± 0.2	0.3 [0.05–0.6]* (0.4)	

Data are reported as mean ± SD or [95% confidence level]; and (Effect size with “Cohen´d”)

* Indicates statistically significant intragroup differences (p<0.05)

** Indicates statistically significant intragroup differences (p<0.001)

† Indicates statistically significant between-groups differences (p<0.05)

†† Indicates statistically significant between-groups differences (p<0.001)

**Fig 4:**
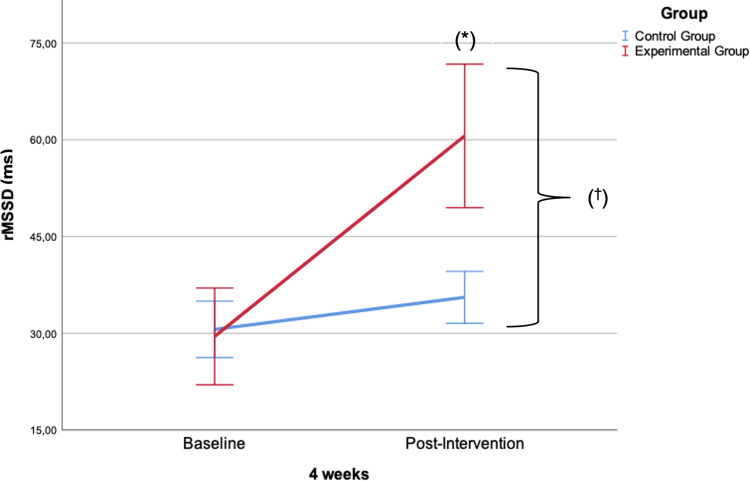
Intra-groups and between-groups mean changes post-intervention in rMSSD (ms). Statistically significant intragroup differences: *(p<0.001). Statistically significant between-groups differences: ^†^ (p<0.001).

Table 2 includes a comparison between groups, which showed statistically significant differences (p<0.05) and moderate effect sizes (*d*≥0.6) for NPRS, mean HR, pNN50, rMSSD (Fig 4), SD1, RMS, PCS and TSK-11 values all in favour of the IG.

Analysis of variance for repeated measures, within the group (intervention group vs. control group) like the between-subjects factor, and time effects (baseline vs post-intervention) as the within-subjects factor, which showed statistically significant differences, were found to favor the IG in the intensity of pain perceived (NPRS): F_1, 29_ = 328.56 (p< 0.001) η^2^ = 0.92; catastrophizing (PCS): F_1, 29_ = 229.34 (p<0.001) η^2^ = 0.88 and kinesiophobia (TSK-11): F_1, 29_ = 868.97 (p< 0.001) η^2^ = 0.97. RMS measured during MVC showed statistically significant changes (p<0.001) for the UT and SCM muscles right and left: RMS UT Right (μV): F_1, 29_ = 1733.70 (p< 0.001) η^2^ = 0.98; RMS UT Left (μV): F_1, 29_ = 566.17 (p< 0.001) η^2^ = 0.95; RMS SCM Right (μV): F_1, 29_ = 124.14 (p< 0.001) η^2^ = 0.81; RMS SCM Leftt (μV): F_1, 29_ = 155.97 (p< 0.001) η^2^ = 0.84.

With respect to HRV, there were statistically significant changes in Mean HR (bpm): F_1, 29_ = 1678.49 (p< 0.001) η^2^ = 0.98; pNN50 (%): F_1, 29_ = 90.60 (p< 0.001) η^2^ = 0.75; rMSSD (ms): F_1, 29_ = 416.10 (p< 0.001) η^2^ = 0.93; LF (ms2): F_1, 29_ = 153.92 (p< 0.001) η^2^ = 0.84; HF (ms2): F_1, 29_ = 160.82 (p< 0.001) η^2^ = 0.85; LF/HF ratio: F_1, 29_ = 116.86 (p< 0.001) η^2^ = 0.80; SD1 (ms): F_1, 29_ = 599.81 (p< 0.001) η^2^ = 0.95; SD2 (ms): F_1, 29_ = 325.41 (p< 0.001) η^2^ = 0.92; SS (ms): F_1, 29_ = 335.55 (p< 0.001) η^2^ = 0.92; S/PS ratio: F_1, 29_ = 79.84 (p< 0.001) η^2^ = 0.73.

## Discussion

The aim of this pilot study was to investigate the effects of a multimodal physiotherapy intervention on psychophysiological parameters in fighter pilots with FRNP. The main results showed significant improvements (decrease in NPRS and an increase in HRV) in comparison to the group that did not receive treatment. These findings suggest that a multimodal physiotherapy programme based on supervised neck exercises with laser-guided feedback and ICT may have the potential to influence short-term autonomic balance in fighter pilots with FRNP. The occupational stressors and risk factors recently described for fighter pilots [1, 41] could position these therapeutic interventions as work-related occupational health strategies [8, 42].

The psychophysiological changes shown could also be influenced by the environment in which the interventions took place. Recently, Morera-Balaguer et al. [43] indicated the need for rehabilitation professionals to review factors associated with patient-centred care. In this sense, participants had easy and convenient access to the place where the interventions were developed in their work environment. According to these authors, the environment where the physiotherapy programme takes place determines the quality perceived by the user. The fact that the participants in the present study received the intervention in the same ambient conditions where they carry out their work could therefore have had an effect on the results achieved.

Perceived neck pain intensity was also significantly reduced following the intervention (Table 2), surpassing the MDC and MCID [30]. The reduction in neck pain intensity could be attributed to the hypoalgesic effects derived from the combination of exercise and ICT [25]; the activation of endogenous inhibitory analgesic mechanisms (related to SNC and SNA) could be enhanced by combining the isolated effects of ICT [25, 30] and neck specific exercise [44].

Positive effects were achieved after 4 weeks of an intervention, unlike those reported by Yesil et al. [45] who detected significant changes after six weeks of ICT or TENS combined with neck stabilization exercises. The average age and work activity of the pilots (25 vs 40 years) may have influenced their ability to learn the exercises, where the focus of attention (supervision guided by laser feedback) facilitated optimal performance [21, 26]. Regarding exercise frequency, these authors consider that a series of 8–10 repetition trials with an external focus is sufficient to improve motor learning.

The changes in HRV showed an increase in autonomic balance, characterised by an increase in parasympathetic activity and a reduction in sympathetic activity [29]. Our results are similar to those observed by Espejo-Antúnez et al. [14] when ICT was applied in isolation. The authors reported an increase in parasympathetic activity which was very similar to that observed in the present study.

The results also revealed a moderate increase of the myoelectric activity of the sternocleidomastoid and upper trapezius (*d* = 0.6) (Table 2). This increase has previously been interpreted in military pilots with FRNP [46] as an improvement in muscle function. In this sense, the improvement of electromyographic parameters has been related to clinically relevant changes in perceived pain [47]. Although it should be noted that we have compared absolute values of EMG amplitude which can be affected by a number of factors such as the extent of skin preparation and cross-talk and this may have differed between measurement sessions.

Levels of pain catastrophizing and kinesiophobia also significantly reduced following the intervention, with moderate effect sizes for both variables (d = 0.6) and differences of means similar to the MDC described for musculoskeletal pain [48, 49]. Within-group differences for GI were slightly lower than those reported by Caña-Pino et al. [50] in people with chronic low back pain. These authors conducted the intervention in an academic setting, with 2 sessions per week for 8 weeks. They highlighted the potential benefits of using supervised exercise with laser-guided feedback on the back to control potentially painful movements. In contrast, the multimodal programme proposed in this study was implemented within the FRNP work environment. This clinical entity is closely related to occupational stressors [3, 4]. The fact that the pilot’s attention is focused on the results of their movements rather than the actual movement [21, 26] could partially explain the benefits observed for both variables in work-related musculoskeletal pain. Future studies are needed to assess the clinical benefits of increasing the number of sessions of this multimodal physiotherapy programme.

### Clinical implications

A fighter pilot’s work activity is highly dynamic and adaptable to the demands of flight, as reflected in HRV [1]. The psychophysiological responses observed after a 4 week physiotherapy intervention could have a protective effect and mitigate the impact of FRNP, optimising flight performance and safety. Nevertheless, the preliminary findings reported in this pilot trial need to be corroborated in future studies.

### Study limitations

There are some methodological limitations that need to be considered when interpreting the current results. Firstly, all pilots use the same aircraft model (F-5), therefore the results cannot be extrapolated to pilots flying other fighter aircrafts. Secondly, the measurements were taken within 48 hours of the last flight, and there may be variations during the assessment time frame. Longer-term follow-up is needed to validate the stability of these findings and to assess possible changes over time. Finally, the sample size is limited since this was a pilot trial and future research is therefore required to corroborate these findings.

## Conclusions

Four weeks of a multimodal physiotherapy program based on neck specific supervised ELGF and ICT facilitated an improved autonomic balance, reduced perceived pain intensity, improved muscle activation and reduced catastrophizing and kinesiophobia in fighter pilots with FRNP. However, the current findings are based only on a convenience sample collected in a Spanish population. Future studies with larger sample sizes and implemented in other geographical areas are needed to confirm the efficacy of the proposed intervention.

## Supporting information

S1 Checklist CONSORT 2010 checklist of information to include when reporting a randomised trial*. (DOC)

S1 Appendix Supervised neck specific exercises with laser-guided feedback. (DOCX)

S2 Appendix Interferential current electromassage sequence. (DOCX)

S1 File (DOCX)
