# Supplementary material for: Psychophysiological responses to a multimodal physiotherapy program in fighter pilots with flight-related neck pain: A pilot trial
Source: PLoS One. 2024 Jul 5;19(7):e0306708. doi: 10.1371/journal.pone.0306708 (PMC11226082; doi:10.1371/journal.pone.0306708)
Supplement: S2 Appendix — (DOCX) [file pone.0306708.s003.docx]

**Supplementary Appendix 2. Interferential current electromassage sequence**

Interferential current electro-massage (ICE) is a technique which combines simultaneously manual therapy (massage) and interferential current therapy^13^. ICE was performed in the neck region for 15 minutes. We used a current bipolar mode, using a carrier frequency of 4000 Hz at constant voltage and an amplitude-modulated frequency of 100 Hz (Sonopuls 692®; Enraf-Nonius BV, Rotterdam, The Netherlands), was administered. The physiotherapist who performed the intervention, performed the sequence of manual soft tissue therapy while administering the interferential current into the body through the skin^13^ by the neck, shoulder and scapular areas. Sponges were used which measured 8 x 12 cm and were moistened with hot water. The intensity was set to provide a strong and comfortable tingling, without evoking muscle twitches even though a slight vibration (fasciculation) was allowed.

The sequence combined (A) superficial stroke over the neck-shoulder for 30-45 seconds; (B) deep sliding movements, alone or (C) combined with shoulder drop, for 4-5 minutes; (D) bilateral kneading of the upper trapezius (4-5 minutes); (E) slight stretching of cervical muscles (upper trapezius, sternocleidomastoid, and levator scapulae); and repetition of step (A):


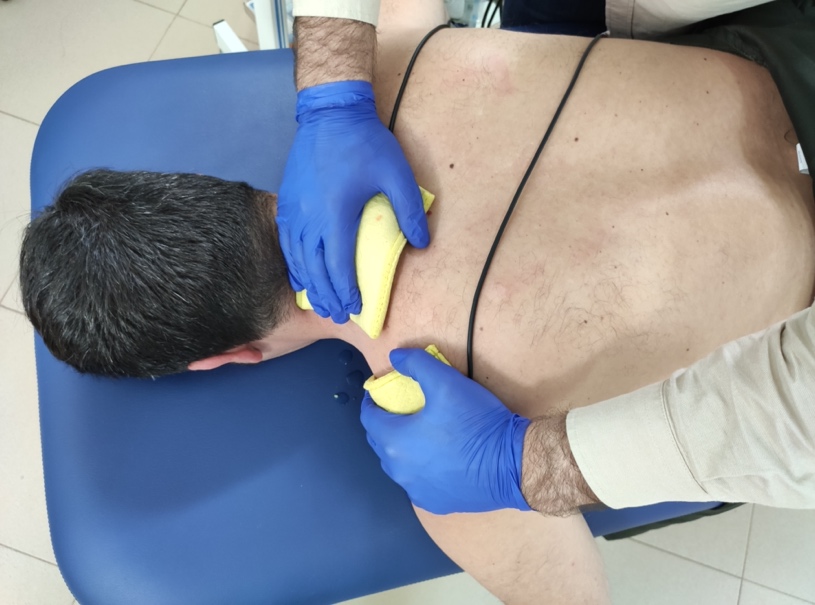

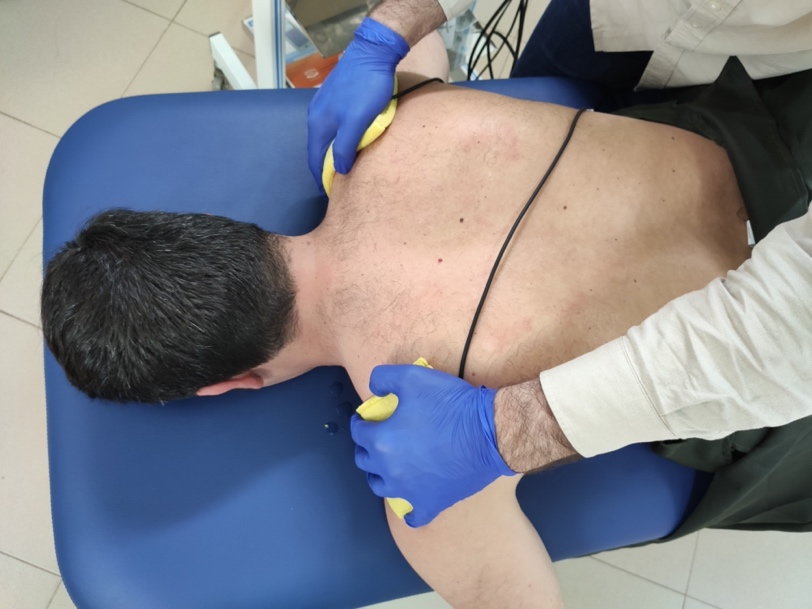

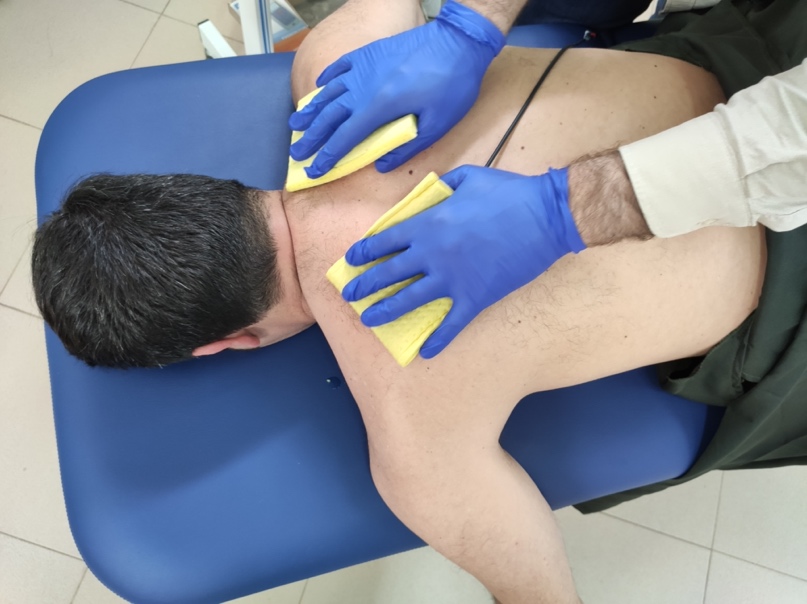

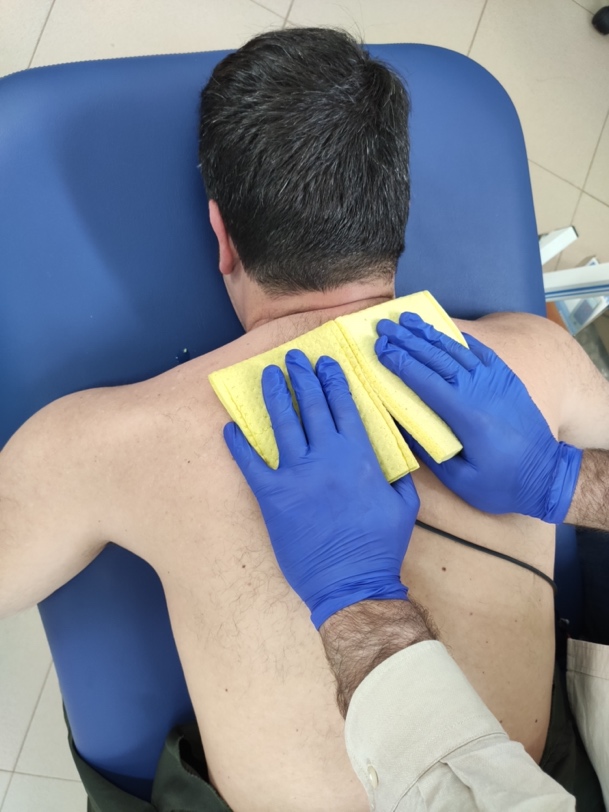


(D) Bilateral kneading of the upper trapezius.

(C) Deep sliding movements combined with shoulder drop.

(B) Deep sliding movements (alone).

(A) Superficial stroke over the neck-shoulder.


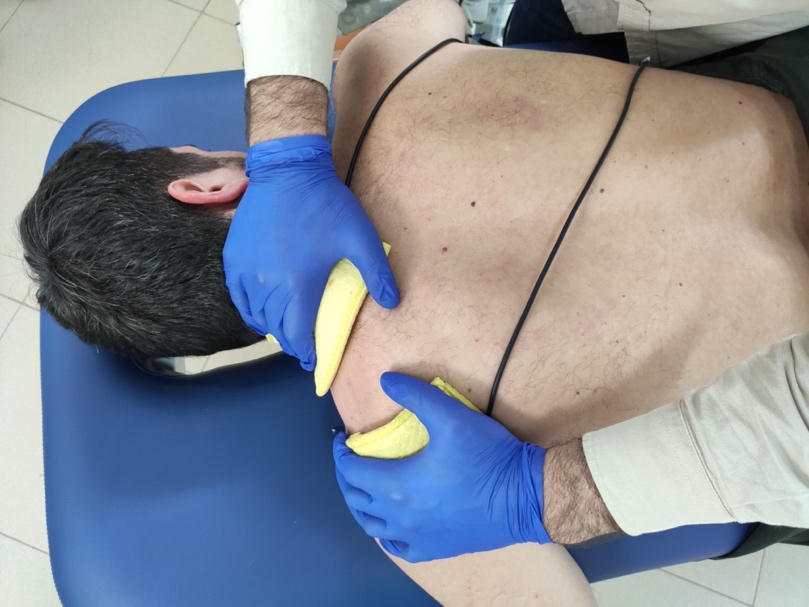

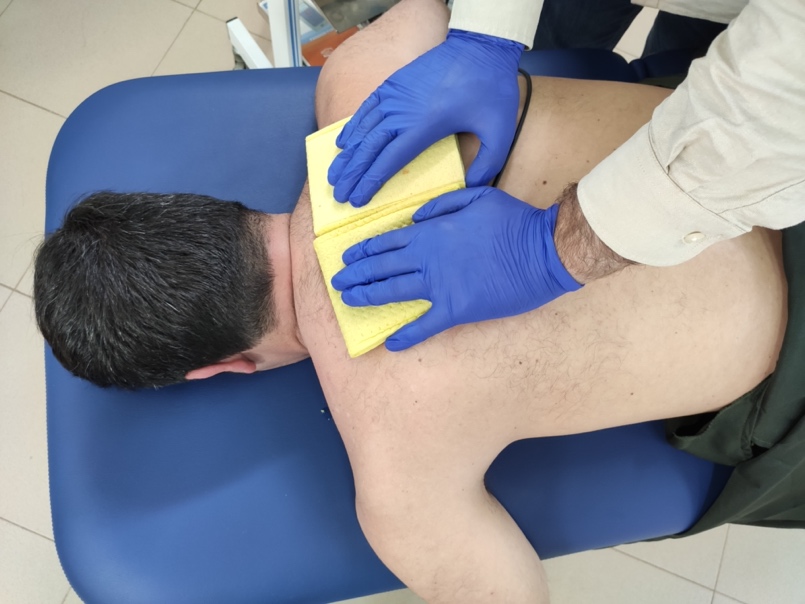


**Figure 1. Sequence for performing Interferential Current Electromassage (ICE).**

(D) Slight stretching of cervical muscles.

(E) Superficial stroke as in step (A).
