# Supplementary material for: Psychophysiological responses to a multimodal physiotherapy program in fighter pilots with flight-related neck pain: A pilot trial
Source: PLoS One. 2024 Jul 5;19(7):e0306708. doi: 10.1371/journal.pone.0306708 (PMC11226082; doi:10.1371/journal.pone.0306708)
Supplement: S1 File — (DOCX) [file pone.0306708.s004.docx]

**A research proposal entitled**

**ASSESSMENT AND APPROACH TO FLIGHT-RELATED NECK PAIN IN FIGHTER PILOTS FROM A BIOPSYCHOSOCIAL APPROACH**

Principal Investigator: Carlos Fernández Morales

Summary

[1 INTRODUCTION 3](#_Toc156941515)

[2 OBJECTIVES 5](#_Toc156941516)

[2.1 General objectives 5](#_Toc156941517)

[2.2 Specific objectives 5](#_Toc156941518)

[3 METHODS 6](#_Toc156941519)

[3.1 Study design 6](#_Toc156941520)

[3.2 Participants 6](#_Toc156941521)

[3.3 Outcome measures 7](#_Toc156941522)

[**3.3.1 Neck Disability Index 7**](#_Toc156941523)

[**3.3.2 Catastrophizing Pain 8**](#_Toc156941524)

[**3.3.3 Kinesiophobia 9**](#_Toc156941525)

[**3.3.4 Myoelectric activity 9**](#_Toc156941526)

[**3.3.5 Outcome Measures of Heart Rate Variability 11**](#_Toc156941527)

[3.4 Intervention 13](#_Toc156941528)

[**3.4.1 Cervical Laser guided exercise therapy (LGTE) 13**](#_Toc156941529)

[**3.4.2 Interferential current electromassage 14**](#_Toc156941530)

[3.5 Sample size 15](#_Toc156941531)

[3.6 Statistical Analysis 16](#_Toc156941532)

[4 INFORMED CONSENT FORM 17](#_Toc156941533)

[5 REFERENCES 20](#_Toc156941534)

# INTRODUCTION

Neck pain represents a major health problem, with a high socio-economic impact^1^. Among the working population, its annual prevalence varies between 30 and 50%^2^, increasing significantly in fighter pilots^3^. According to NATO, flight-related neck pain was estimated to cost up to 7 man-years of lost productivity in fighter pilots, drastically reducing the return on investment in pilot training, estimated at more than 2 million euros^4^. Its multifactorial etiology has contributed to the consideration of certain occupational exposures (physical and psychosocial) as causal factors related to absenteeism from work^5,6^ in the general population, and to flight safety in the face of physical demands by G-forces in fighter pilots^3–5^.

Flight-related neck pain has been defined as a clinical entity specific to military pilots^4^. It refers to the significant neck pain that occurs during or within 48 hours after the flight. It does not refer to pain that is obviously due to other activities or causes. Previous studies have identified mobility limitations in the cervical region, force limitations and cervical impairment in fighter pilots who suffer from flight-related neck pain^3,7,8^. Over the last decade, investment in technological development by national defense administrations has transformed military aviation and, consequently, the training of fighter pilots. However, strategies aimed at reducing the direct and indirect costs arising from work-related musculoskeletal disorders in fighter pilots are limited and not very clarifying^4^.

Current evidence-based guidelines for the management of neck pain highlight the importance of multimodal physiotherapy programs based on the interaction between techniques such as soft tissues mobilization, exercise and electrical stimulation^4,9,10^. The benefits of combining both interventions lie in the possible effects on the impairments of flexibility of key muscles related to the lower cervical and upper thoracic spine^4,10^ The combination of interferential current with other therapeutic modalities has been investigated on neck pain (plus supervised exercise)^9^, low back pain (plus manual therapy)^11,12^ or shoulder pain (simultaneously interferential current plus manual therapy)^13^. Nevertheless, the results are still uncertain^14^. Recently, NATO has recommended electrical stimulation as first-choice treatment in fighter pilots who suffer from flight-related neck pain^4^. However, it is unknown what effect may electrical stimulation add in conjunction with simultaneous application of manual therapy (electro-massage) in the neck region of fighter pilots.

The clinical efficacy of the cervical coordination exercises versus protocols that only include strength or endurance in reducing pain has also been analysed^15^. In recent years, the development of rehabilitation technologies has reported differences between types of exercise according to whether the exercise requires the individual to focus on their body while moving a body region (exercise with an internal locus of movement control) or to focus on clues provided by the environment, with the effect being achieved after movement has been performed (exercise with an external locus). Previous studies about neck pain have reported improvements in motor skill learning when the type of exercise was guided by means of an external locus located in the head (eg. Laser pointer)^16^. The clinical utility has been explained as facilitating the establishment of effective neural connections that optimize exercise performance^17^.

Notwithstanding, there is no clear consensus on which is the most effective approach for fighter pilots with flight-related neck pain.

The aim of this study will be to analyse the immediate effects of a 4-week multimodal physiotherapy program which combines cervical supervised exercises with laser-guided feedback (ELGF) and interferential current electro massage (ICE) in fighter pilots with flight-related neck pain.

# OBJECTIVES

## General objectives

1. To assess cervical status and design a reliable assessment protocol based on the biopsychosocial model in military pilots with flight-related neck pain.

2. To analyse the relationship between cognitive demand and stress in each flight segment using Heart Rate Variability (HRV) in a flight simulator.

3. To develop and analyse the impact of a multimodal physiotherapy programme on clinimetric and psychophysiological variables in fighter pilots with flight-related neck pain.

## Specific objectives

1. To assess the degree of disability, limitation of cervical mobility, perceived pain, cervical repositioning and electromyographic disturbances and degree of kinesiophobia and catastrophism in fighter pilots with flight-related neck pain.

2. To find out the possible associations according to age and flight experience with the clinimetric variables assessed.

3. To analyse the VFC in the take-off, flight and landing segments during emergency manoeuvres in the flight simulator.

4. To determine what changes occur during flight and which segment produces the greatest stress response in pilots.

5. Design and implement a multimodal physiotherapy programme combining supervised cervical exercise with laser-guided feedback (ELGF) and interferential current electro-massage (ICE).

6. To analyse the impact of the multimodal physiotherapy programme on variables analysed in the designed assessment protocol and on physiological changes using HRV.

# METHODS

## Study design

We propose to conduct a single-blind randomised clinical trial with concealed allocation, intention-to-treat analysis and in which the treatment allocation is not known to the evaluator and the statistical investigator. The study will be conducted in accordance with the recommendations of the CONSORT^18^. statements. The future study will be conducted in accordance with the Declaration of Helsinki.

All data will be collected in the same room, at a temperature of 22–25 °C. Always in the same order and under the same environmental conditions for all subjects, both before and after the intervention.

## Participants

Convenience sampling will be followed. The inclusion criteria will be: (i) fighter pilots (male and female) who, at the time of the assessment, are an instructor or student attached to the 23rd Wing of the Talavera Air Base, Air Force (FAE), Badajoz; (ii) fighter pilots diagnosed with flight-related neck pain according to the International Classification proposed by a NATO expert panel4; (iii) fighter pilots diagnosed with flight-related neck pain according to the International Classification proposed by a NATO expert panel; (iv) fighter pilots diagnosed with flight-related neck pain according to the International Classification proposed by a NATO expert panel5; a minimum perceived pain score of 3/10 on the Numerical Pain Rating Scale (NPRS) on the early morning assessment within 48 hours of the last flight; (iv) scores of ≥5 points on the Neck Disability Index (NDI), and a cervical positioning error of ≥4.5°^19^. Exclusion criteria shall be: (i) Personal Psychological Apprehension Scale (PPAS) score greater than 37.5^20^; (ii) contraindication to electrical stimulation; (iii) having received physiotherapy or any other routine medical care six weeks prior to data collection; (iv) any regular use of medications known to affect autonomic nervous system (ANS) function or pain perception, including opioids, antidepressants, benzodiazepines, anti-inflammatory drugs and beta-blockers, two weeks prior to participation in this study; and (v) being involved in ongoing medico-legal disputes.

- - 1. Numeric Pain Rating Scale

Numeric Pain Rating Scale (NPRS) is a 11-point numeric rating scale, where 0 denotes “no pain” and 10 denotes “the maximum bearable pain”. The minimum clinically important difference (MCID) for this tool have been established at 1.3 points and the minimum detectable change (MDC) at 2.1 points^9^, in individuals with neck pain. The NPRS is a valid scale with moderate test-retest reliability in this population (ICC: 0.76, 95% CI 0.51 to 0.87)^9,21^.

## Outcome measures

### Neck Disability Index

Cervical Disability will be evaluated using the validated Spanish version of the Neck Disability Index (NDI)^22^. [This](https://www.collinsdictionary.com/es/diccionario/ingles-espanol/this) [questionnaire](https://www.collinsdictionary.com/es/diccionario/ingles-espanol/questionnaire) [consists](https://www.collinsdictionary.com/es/diccionario/ingles-espanol/consist) [of](https://www.collinsdictionary.com/es/diccionario/ingles-espanol/of) 10 [sections](https://www.collinsdictionary.com/es/diccionario/ingles-espanol/section), 4 [of](https://www.collinsdictionary.com/es/diccionario/ingles-espanol/of) [them](https://www.collinsdictionary.com/es/diccionario/ingles-espanol/them) [are](https://www.collinsdictionary.com/es/diccionario/ingles-espanol/are) [related](https://www.collinsdictionary.com/es/diccionario/ingles-espanol/related_2) [to](https://www.collinsdictionary.com/es/diccionario/ingles-espanol/to) [subjective](https://www.collinsdictionary.com/es/diccionario/ingles-espanol/subjective) [symptoms](https://www.collinsdictionary.com/es/diccionario/ingles-espanol/symptom) (pain [intensity](https://www.collinsdictionary.com/es/diccionario/ingles-espanol/intensity), [headache](https://www.collinsdictionary.com/es/diccionario/ingles-espanol/headache), [ability](https://www.collinsdictionary.com/es/diccionario/ingles-espanol/ability) [to](https://www.collinsdictionary.com/es/diccionario/ingles-espanol/to) [concentrate](https://www.collinsdictionary.com/es/diccionario/ingles-espanol/concentrate) [and](https://www.collinsdictionary.com/es/diccionario/ingles-espanol/and) [sleep](https://www.collinsdictionary.com/es/diccionario/ingles-espanol/sleep) quality) [and](https://www.collinsdictionary.com/es/diccionario/ingles-espanol/and) [the](https://www.collinsdictionary.com/es/diccionario/ingles-espanol/the) [other](https://www.collinsdictionary.com/es/diccionario/ingles-espanol/other) 6 [are](https://www.collinsdictionary.com/es/diccionario/ingles-espanol/are) [related](https://www.collinsdictionary.com/es/diccionario/ingles-espanol/related_2) [to](https://www.collinsdictionary.com/es/diccionario/ingles-espanol/to) [basic](https://www.collinsdictionary.com/es/diccionario/ingles-espanol/basic) [activities](https://www.collinsdictionary.com/es/diccionario/ingles-espanol/activity) [of](https://www.collinsdictionary.com/es/diccionario/ingles-espanol/of) [daily](https://www.collinsdictionary.com/es/diccionario/ingles-espanol/daily) [life](https://www.collinsdictionary.com/es/diccionario/ingles-espanol/life) (personal [care](https://www.collinsdictionary.com/es/diccionario/ingles-espanol/care), [ability](https://www.collinsdictionary.com/es/diccionario/ingles-espanol/ability) [to](https://www.collinsdictionary.com/es/diccionario/ingles-espanol/to) [lift](https://www.collinsdictionary.com/es/diccionario/ingles-espanol/lift) [weights](https://www.collinsdictionary.com/es/diccionario/ingles-espanol/weight), [reading](https://www.collinsdictionary.com/es/diccionario/ingles-espanol/reading), [work](https://www.collinsdictionary.com/es/diccionario/ingles-espanol/work), [driving](https://www.collinsdictionary.com/es/diccionario/ingles-espanol/driving), [leisure](https://www.collinsdictionary.com/es/diccionario/ingles-espanol/leisure) [activities](https://www.collinsdictionary.com/es/diccionario/ingles-espanol/activity) [and](https://www.collinsdictionary.com/es/diccionario/ingles-espanol/and) [leisure](https://www.collinsdictionary.com/es/diccionario/ingles-espanol/leisure) time). NDI has been shown to be valid and reliable for measuring pain and cervical disability (Cronbach Alpha: 0,944; ICC: 0.88, 95% CI 0.80 to 0.93)^21,23^. It is he most used scale for neck pain and disability, having been previously used [in](https://www.collinsdictionary.com/es/diccionario/ingles-espanol/in_1) fighter pilots^8^. The MCID has been reported in 7 points^21^ and MDC at 9.8 points^9^.

Each item of this self-completed questionnaire presents six possible answers. Scores range from 0 (no disability) to 5 (complete disability) points. The total score (maximum 50 points) is calculated by adding up the responses for each item. [The](https://www.collinsdictionary.com/es/diccionario/ingles-espanol/the) [interpretation](https://www.collinsdictionary.com/es/diccionario/ingles-espanol/interpretation) [of](https://www.collinsdictionary.com/es/diccionario/ingles-espanol/of) [the](https://www.collinsdictionary.com/es/diccionario/ingles-espanol/the) [questionnaire](https://www.collinsdictionary.com/es/diccionario/ingles-espanol/questionnaire) is classified as follows: "no disability" (NDI < 10% of maximum total score), "mild" (NDI between 10% and < 30%), "moderate" (NDI between 30% and < 50%), "severe" (NDI between 50% and < 70%) or "very severe" (NDI >= 70%) degree of disability.

### Catastrophizing Pain

Catastrophizing is a cognitive factor that involves an exaggerated negative appraisal toward pain stimuli and pain experience^24^. Pain Catastrophizing Scale (PCS) [is](https://www.collinsdictionary.com/es/diccionario/ingles-espanol/is_1) [a](https://www.collinsdictionary.com/es/diccionario/ingles-espanol/a_1) [self](https://www.collinsdictionary.com/es/diccionario/ingles-espanol/self_1)-[administered](https://www.collinsdictionary.com/es/diccionario/ingles-espanol/administered) [scale](https://www.collinsdictionary.com/es/diccionario/ingles-espanol/scale) (Likert scale) [of](https://www.collinsdictionary.com/es/diccionario/ingles-espanol/of) 13 [items](https://www.collinsdictionary.com/es/diccionario/ingles-espanol/item) [and](https://www.collinsdictionary.com/es/diccionario/ingles-espanol/and) [one](https://www.collinsdictionary.com/es/diccionario/ingles-espanol/one_1) [of](https://www.collinsdictionary.com/es/diccionario/ingles-espanol/of) [the](https://www.collinsdictionary.com/es/diccionario/ingles-espanol/the) [most](https://www.collinsdictionary.com/es/diccionario/ingles-espanol/most_2) [used](https://www.collinsdictionary.com/es/diccionario/ingles-espanol/used) and reliable (Cronbach [alpha](https://www.collinsdictionary.com/es/diccionario/ingles-espanol/alpha): 0.79)^25^ [to](https://www.collinsdictionary.com/es/diccionario/ingles-espanol/to) [assess](https://www.collinsdictionary.com/es/diccionario/ingles-espanol/assess) [the](https://www.collinsdictionary.com/es/diccionario/ingles-espanol/the) catastrophizing [of](https://www.collinsdictionary.com/es/diccionario/ingles-espanol/of) [pain](https://www.collinsdictionary.com/es/diccionario/ingles-espanol/pain). [In](https://www.collinsdictionary.com/es/diccionario/ingles-espanol/in_1) [it](https://www.collinsdictionary.com/es/diccionario/ingles-espanol/it), [subjects](https://www.collinsdictionary.com/es/diccionario/ingles-espanol/subject) [refer](https://www.collinsdictionary.com/es/diccionario/ingles-espanol/refer) [to](https://www.collinsdictionary.com/es/diccionario/ingles-espanol/to) [their](https://www.collinsdictionary.com/es/diccionario/ingles-espanol/their) [past](https://www.collinsdictionary.com/es/diccionario/ingles-espanol/past) [painful](https://www.collinsdictionary.com/es/diccionario/ingles-espanol/painful) [experiences](https://www.collinsdictionary.com/es/diccionario/ingles-espanol/experience) [and](https://www.collinsdictionary.com/es/diccionario/ingles-espanol/and) [indicate](https://www.collinsdictionary.com/es/diccionario/ingles-espanol/indicate) [the](https://www.collinsdictionary.com/es/diccionario/ingles-espanol/the) [degree](https://www.collinsdictionary.com/es/diccionario/ingles-espanol/degree) [to](https://www.collinsdictionary.com/es/diccionario/ingles-espanol/to) [which](https://www.collinsdictionary.com/es/diccionario/ingles-espanol/which) [they](https://www.collinsdictionary.com/es/diccionario/ingles-espanol/they) [experienced](https://www.collinsdictionary.com/es/diccionario/ingles-espanol/experienced) [each](https://www.collinsdictionary.com/es/diccionario/ingles-espanol/each) [of](https://www.collinsdictionary.com/es/diccionario/ingles-espanol/of) [the](https://www.collinsdictionary.com/es/diccionario/ingles-espanol/the) 13 [thoughts](https://www.collinsdictionary.com/es/diccionario/ingles-espanol/thought) [or](https://www.collinsdictionary.com/es/diccionario/ingles-espanol/or) [feelings](https://www.collinsdictionary.com/es/diccionario/ingles-espanol/feeling). [The](https://www.collinsdictionary.com/es/diccionario/ingles-espanol/the) [score](https://www.collinsdictionary.com/es/diccionario/ingles-espanol/score) [ranges](https://www.collinsdictionary.com/es/diccionario/ingles-espanol/range_2) [from](https://www.collinsdictionary.com/es/diccionario/ingles-espanol/from) 0 (never) [to](https://www.collinsdictionary.com/es/diccionario/ingles-espanol/to) 4 (always).

Total score [reflects](https://www.collinsdictionary.com/es/diccionario/ingles-espanol/reflect) [the](https://www.collinsdictionary.com/es/diccionario/ingles-espanol/the) [level](https://www.collinsdictionary.com/es/diccionario/ingles-espanol/level) [of](https://www.collinsdictionary.com/es/diccionario/ingles-espanol/of) catastrophism obtained [of](https://www.collinsdictionary.com/es/diccionario/ingles-espanol/of) [the](https://www.collinsdictionary.com/es/diccionario/ingles-espanol/the) subject's [pain](https://www.collinsdictionary.com/es/diccionario/ingles-espanol/pain) is obtained (higher score means worse result).

### Kinesiophobia

[The](https://www.collinsdictionary.com/es/diccionario/ingles-espanol/the) [Spanish](https://www.collinsdictionary.com/es/diccionario/ingles-espanol/spanish) [version](https://www.collinsdictionary.com/es/diccionario/ingles-espanol/version) [of](https://www.collinsdictionary.com/es/diccionario/ingles-espanol/of) [the](https://www.collinsdictionary.com/es/diccionario/ingles-espanol/the) Tampa Kinesiophobia [Scale](https://www.collinsdictionary.com/es/diccionario/ingles-espanol/scale) (TSK-11) [will be](https://www.collinsdictionary.com/es/diccionario/ingles-espanol/was) [used](https://www.collinsdictionary.com/es/diccionario/ingles-espanol/used)^26^. [This](https://www.collinsdictionary.com/es/diccionario/ingles-espanol/this) subjective [questionnaire](https://www.collinsdictionary.com/es/diccionario/ingles-espanol/questionnaire) [contains](https://www.collinsdictionary.com/es/diccionario/ingles-espanol/contain) 11 [items](https://www.collinsdictionary.com/es/diccionario/ingles-espanol/item) [designed](https://www.collinsdictionary.com/es/diccionario/ingles-espanol/design) [to](https://www.collinsdictionary.com/es/diccionario/ingles-espanol/to) [assess](https://www.collinsdictionary.com/es/diccionario/ingles-espanol/assess) [the](https://www.collinsdictionary.com/es/diccionario/ingles-espanol/the) patient's [fear](https://www.collinsdictionary.com/es/diccionario/ingles-espanol/fear) [of](https://www.collinsdictionary.com/es/diccionario/ingles-espanol/of) [moving](https://www.collinsdictionary.com/es/diccionario/ingles-espanol/moving) [and](https://www.collinsdictionary.com/es/diccionario/ingles-espanol/and) [re](https://www.collinsdictionary.com/es/diccionario/ingles-espanol/re_1)-injury. [Score](https://www.collinsdictionary.com/es/diccionario/ingles-espanol/score) [ranges](https://www.collinsdictionary.com/es/diccionario/ingles-espanol/range_2) [from](https://www.collinsdictionary.com/es/diccionario/ingles-espanol/from) 11 [to](https://www.collinsdictionary.com/es/diccionario/ingles-espanol/to) 44 [points](https://www.collinsdictionary.com/es/diccionario/ingles-espanol/point). Each item is associated with a 4-point Likert scale (1= “strongly disagree”,4= “strongly agree”). Higher scores correspond with a greater fear of pain, movement and injury. The Spanish version of the TSK-11 has shown good reliability and validity (Cronbach [alpha](https://www.collinsdictionary.com/es/diccionario/ingles-espanol/alpha): 0.79)^26^. [A](https://www.collinsdictionary.com/es/diccionario/ingles-espanol/a_1) [higher](https://www.collinsdictionary.com/es/diccionario/ingles-espanol/higher) [score](https://www.collinsdictionary.com/es/diccionario/ingles-espanol/score) [indicates](https://www.collinsdictionary.com/es/diccionario/ingles-espanol/indicate) [higher](https://www.collinsdictionary.com/es/diccionario/ingles-espanol/higher) [levels](https://www.collinsdictionary.com/es/diccionario/ingles-espanol/level) [of](https://www.collinsdictionary.com/es/diccionario/ingles-espanol/of) kinesiophobia.

### Myoelectric activity

Myoelectric activity will be measured with surface electromyography (sEMG) according to the recommendations of the NATO expert panel^4^. A protocol similar to that established in previous studies will be used^27,28^. The sEMG recordings will be taken using mDurance® system (mDurance Solutions SL, Granada, Spain). It is a portable and low-costs EMG system, which has proved to be a valid tool to measure muscle activity during dynamic contractions^29^. The system consists of a Shimmer3 EMG unit (Realtime Technologies Ltd, Dublin, Ireland), a bipolar sEMG sensor for the acquisition of superficial muscle activity. Each Shimmer sensor is composed of two sEMG channels, which register the electrical signal via bluetooth with a sampling rate at 1,024 Hz. Shimmer applies a bandwidth 8.4 kHz the EMG signal resolution is 24 bits and overall amplification of 100–10,000 V/V.

As sEMG recommended for the noninvasive assessment of muscles SENIAM^30^, the electrodes placement areas will be shaved if necessary, and disinfected with 70% alcohol. Next, five disposable, bipolar, self-adhesive electrodes with pre-gelled surface discs and an active diameter with dimensions 41 mm × 21 mm, diameter 10 mm (Ag/AgCl, Blue Sensor N-00-S, Medicotest A/S, Olstykke, Denmark) will be placed pairwise (with an interelectrode distance of 21 ± 1 mm), on the muscle belly parallel to the muscle fibers. Myoelectric activity will be recorded from two muscles bilaterally: the anterior neck (the lower part of the sternocleidomastoid muscle belly), and the upper shoulders (upper trapezius muscle bellies, midway between the occiput and the acromion).

A reference electrode will be placed around the bone rim of the acromion.

Firstly, participants will be instructed through pre-registration tests before myoelectric activity recordings will be made. Pre-recording tests consisted of the subject pushing their heads against manual resistance in extension, as indicated by Pousette et al^27^. Subjects will be comfortably seated in the gondola’s flight seat, with their back against the seat and their trunk and shoulders fixed to a rigid seat back using two straps placed along both shoulders, and the elbows with a flexion of 60º approximately. The participants will be placed in a neutral cranio-cervical position when they will be instructed to push with increasing force up to maximal voluntary force and hold for 3 seconds in order to avoid injury and minimize the risk of dynamic contractions. Three trials will be done, with 1 minute rest in between each other, in order to enhance maximum voluntary contraction (MVC) stability^27^.

Then, three trials (with a duration of 1 second each attempt) to 15-30% MVC will be performed, [as](https://www.collinsdictionary.com/es/diccionario/ingles-espanol/as) [proposed](https://www.collinsdictionary.com/es/diccionario/ingles-espanol/proposed) [by](https://www.collinsdictionary.com/es/diccionario/ingles-espanol/by) Calamita [et](https://www.collinsdictionary.com/es/diccionario/ingles-espanol/et) [al](https://www.collinsdictionary.com/es/diccionario/ingles-espanol/al)^31^, and the mean of the two highest trials will be defined as MVC.

### Outcome Measures of Heart Rate Variability

Interbeat time interval (R-R) variation will be used to determine the autonomic modulation using Firstbeat Bodyguard equipment (Firstbeat Technologies, Jyväskylä, Finland). This device will be used to record HRV data for 20 min (at rest and during ICT application). Recordings will be exported from the devices to the computer via Firstbeat Uploader Software (Firstbeat Technologies) and analysed using Kubios Software (University of Eastern Finland, Kuopio, Finland). To calculate the autonomous balance, the HRV method based on the Poincaré plot will be used^32,33^. This software has proven to be extremely valid and capable of recording non-linear trends that are frequently present on R-R intervals of record^34^.

Time-domain variables:

- ***MeanHR (bpm):*** It corresponds to the interval between two beats (R peaks on the ECG).
- ***pNN50 (%):*** Percentage of consecutive RR intervals that differ by more than 50 ms from each other. A high value of pNN50 provides valuable information about high spontaneous heart rate (HR)^35^.
- ***Root Mean Square of the Successive Differences (rMSSD) (ms)***: The square root of the average of the sum of the differences squared between normal adjacent. It shows the degree of activation of the Parasympathetic Nervous System on the cardiovascular system. This parameter reports the short-term variations of the RR intervals. It is directly associated with short-term variability^35,36^.
- ***Min HR and Max HR (bpm)***: They indicate the minimum and maximum heart rate, respectively, using *N* beats.

Frequency-domain variables:

- ***Low-Frequency power (LF) (ms^2^):*** Situated between 0.04 and 0.15 Hz. In long-term recordings, it provides us with more information about the activity of the sympathetic nervous system (SNS)^35^.
- ***High-Frequency power (HF) (ms^2^):*** They are located between 0.15 and 0.4 Hz. HF is clearly related to the activity of the parasympathetic nervous system (PNS) activity and has a relaxation-related effect on HR^35^.
- ***Low/high-Frequency ratio (LF/HF):*** From the low-frequency and high-frequency ratios of the HRV spectral analysis results we can estimate the vagal (related to relaxation and HF) and sympathetic (related to stress and LF) influence. Thus, we can estimate sympathetic-vagal balance^35^.

Non-linear variables:

- ***SD1 (ms):*** Sensitivity of short-term variability of the non-linear range of the HRV. It is considered an indicator of parasympathetic activity^32^.
- ***SD2 (ms)***: Long-term variability of the non-linear range of the HRV. It is a diameter from the Poincaré plot which indicates the degree of longitudinal dispersion. It is thought to reflect long-term changes in RR intervals and it is considered an inverse indicator of parasympathetic activity^32^.
- ***Stress Score (SS) (ms)***: It is an index described by Naranjo-Orellana et al.^32^ to facilitate the physiological interpretation of the Poincaré plot. It is expressed as the inverse of SD2 diameter multiplied by 1000.It is considered directly proportional to the sympathetic activity in the sinus node.
- ***Sympathetic/parasympathetic ratio (S/PS):*** It is also described by Naranjo-Orellana et al.^32^, S/PS is expressed as the quotient of SS and SD1. It is considered to reflect autonomic balance - that is, the relationship between sympathetic and parasympathetic activity.

## Intervention

All intervention procedures will be conducted by the same physical therapist. Los participantes del grupo experimental (n=14) realizaron un programa de LGET based motor control exercises. de Posteriormente, recibieron una sesión de terapia manual combinada con electroterapia ICT denominada electro-masaje^13^. Completaron un total de 8 sesiones en 4 semanas. On the other hand, los participantes del control group (n=17) no recibieron ninguna intervención para determinar el curso natural de la enfermedad. Los sujetos continuaron con sus actividades habituales del vuelo de combate y ejercicio. Se les pidió que no tomasen medicación o buscasen tratamientos alternativos.

### Cervical Laser guided exercise therapy (LGTE)

LGTE is defined as a type of therapeutic exercise that provides external feedback to exercise, achieving an improvement in range of motion and postural control in subjects with spinal pain.

According to Abdollahipour et al. and Chiviacowsky et al. the external focus fosters a kind of automatic control that enables unconscious and rapid control processes^17,37^. In addition, it favours meta-action coupling, which is relevant to the fighter pilot's routine tasks. The "Motion Guidance Clinician Kit" (Motion Guidance LLC, Denver, CO, USA) will be used to carry out the exercise programme. The kit has a panel and a laser guide for the correct execution of cervical movements. The laser shall be positioned by means of an elastic strap at the pilot's forehead. The panel will be placed at a distance of 1.5 metres, attached to the wall. The programme will consist of 4 exercises, which will progress in difficulty according to the tolerance achieved with the passing of the sessions: a) Maintenance of the position of the head (cervical stabilisation); b) Cervical flexion-extension; c) Right-left rotations; d) Right-left latero-flexions. Each exercise consists of 4 sets of 8 repetitions, except the first one, in which the head position is maintained by pointing the laser at the centre of the panel for 30 seconds (4 sets). Between each series there will be 10 seconds of rest. Subjects will start with the subjects seated on a stool, and then move to a standing position from the 3rd session onwards. From the 5th session onwards, the distance between the signals to be reached with the laser will be increased, with the aim of increasing the range of cervical movement in the 3 planes of space. The average time for the complete programme should not exceed 14 minutes. The interventions will be carried out in compliance with the recommendations of the CERT^38^ and TIDIER^39^ statements.

### Interferential current electromassage

Following the motor control exercise programme, interferential current electromassage (ICE) in cervical region will be added for 15 minutes. ICE technique simultaneously combines manual therapy and ICT13. A bipolar application, using a carrier frequency of 4000 Hz at constant voltage and an amplitude-modulated frequency of 100 Hz (Sonopuls 692®; Enraf-Nonius BV, Rotterdam, The Netherlands), will be administered. The physiotherapist in charge of the intervention, in contact with the sponges, performs the sequence of manual soft tissue therapy while administering the interferential current into the body through the skin^13^ in the areas of the neck, shoulders and scapulae. The sequence combines strokes, bilateral lowering of the shoulders and stretching of the neck-shoulder muscles (e.g. upper trapezius and levator scapulae).

A strong level of intensity perception will be reached, but without causing pain or discomfort, as well as obvious muscle contraction, although a slight vibration (fasciculation) was allowed. Two rubber electrodes (6 × 8 cm) inside spontex of equal size were used. The sponges will be moistened with warm water to avoid unpleasant sensations and to allow normal gliding on the skin. Some needles shall be prepared with hot water to moisten the sponges during the procedure, if necessary.

## Sample size

G*power 3.1 software will be used to calculate the sample size required to detect changes in the primary outcome (NPRS scale and JPSE). The calculations will be based on the detection differences of 2.5 points in the NPRS (MCID (minimal clinically important difference) (estimated for a variance in patients with neck pain of 10 points)^40^, assuming a standard deviation (SD) of 2.5 points.

Assuming an effect size (F-test) of 0.27 for ANOVA: repeated measures, within-between interaction groups differences, an alpha level of .05, and power of 80%, a total sample size of 30 participants will be estimated. The sample will be inflated by 10% to account for potential dropouts, resulting in a final target sample size of 34. This calculation showed that a sample size of 17 participants per group will be needed for a confidence interval of 95%, with a power of 80%, assuming a bilateral significance of .05.

## Statistical Analysis

A descriptive analysis will be performed for each of the variables. The normality of the variables will be evaluated using the Shapiro-Wilk test, which showed a normal distribution for all the variables and, thus, parametric tests will be appropriate. Data will be reported as mean # SD. The demographic and clinical variables of the groups at baseline will be compared using the chi-square test for categorical data and the independent-samples t-test for quantitative data. A 2-way repeated measure analysis of variance will be performed to analyse the interaction effects of time (at baseline and post-treatment) in the 2 intervention groups (experimental and control group). The independent and paired-samples t tests will be used for comparisons between and within groups, respectively. Furthermore, the effect size will be calculated through Cohen's d coefficient. A value above 0.8 will be considered high; 0.5 moderate; and lower than 0.2 will be considered low^41^. Significance level will be established at p < 0.05. Data analysis will be made with the statistic software SPSS version 26.0 (SPSS Inc., Chicago, IL, USA).

# INFORMED CONSENT FORM

**Field study**: ASSESSMENT AND APPROACH TO FLIGHT-RELATED NECK PAIN IN FIGHTER PILOTS FROM A BIOPSYCHOSOCIAL APPROACH.

**Patient: Patient ID#:**

**Centre:** Faculty of Medicine **Centre ID#:** UNIVERSITY OF EXTREMADURA

**Researcher:** CARLOS FERNÁNDEZ MORALES **Tutor**: LUIS ESPEJO ANTÚNEZ.

PLEASE READ THE INFORMATION CONTAINED IN THIS DOCUMENT CAREFULLY AND MAKE SURE YOU UNDERSTAND THIS RESEARCH PROJECT. PLEASE SIGN THIS DOCUMENT IF YOU AGREE TO PARTICIPATE IN THIS STUDY. BY YOUR SIGNATURE YOU ACKNOWLEDGE THAT YOU HAVE BEEN INFORMED OF THE PROJECT, ITS REQUIREMENTS AND ITS RISKS AND THAT YOU FREELY AGREE TO PARTICIPATE IN IT.

**PURPOSE OF THE STUDY.**

To participate as a sample subject in a research study to conduct an intervention consisting of current, interferential and supervised cervical exercise with laser-guided feedback in the management of flight-related neck pain.

**PROCEDURES AND DURATION OF THE STUDY.**

The procedure to which you will be subjected will be:

-.Assessment of clinimetric variables (electromyography; assessment of perceived pain (using the NPRS scale); assessment of degrees of involvement (using the PCS and TAMPA scale) and assessment of the ANS during the intervention.

The data will be used exclusively for non-profit research purposes.

**RESULTS OF THE STUDY.**

At the end of the study you will be informed of the overall result of the study if you wish, but NOT of your personal result, which will be treated with total confidentiality in accordance with the Declaration of Helsinki and Law 14/2007, on Biomedical Research.

**RISKS DERIVED FROM PARTICIPATION IN THE STUDY.**

-. THERE ARE NO RISKS ASSOCIATED WITH PARTICIPATION IN THE STUDY.

**BENEFITS.**

Participation in the project will not be financially rewarded. Apart from the above, it is estimated that the development of the study in which you will participate will bring benefits in the improvement of knowledge about the effect of flight on neck pain in fighter and attack pilots, which has not yet been investigated.

**COSTS**. Your participation will be free of charge.

**CONFIDENTIALITY**

In accordance with current legal regulations, the results of the study will be treated with complete confidentiality. The data collection protocol will be archived, and each participant will be assigned a password so that the information obtained cannot be linked to the identity of the subject.

The principal investigator of the project undertakes that the confidentiality of the data that may be obtained in this project will be scrupulously observed, and that the personal data of the participating subjects will not be known by the researchers of the project. In appropriate cases, they will inform the person in charge of medicine, or the persons concerned if they believe that any result of the project could be of interest to them.

For all matters not foreseen in this document, the current legislation on personal data protection will be applied (Law 41/2002, of 14 November, basic law regulating patient autonomy and rights and obligations regarding clinical information and documentation, BOE 274 of 15 November 2002; Organic Law 15/1999, of 13 December, on the Protection of Personal Data; BOE 298 of 14 December 1999; Royal Decree 1720/2007 of 21 December 2007, approving the Regulations for the development of Organic Law 15/1999 of 13 December on the Protection of Personal Data, BOE 17 of 19 January 2008), on biomedical research (Law 14/2007 of 3 July on Biomedical Research; BOE 159 of 4 July 2007) and any other applicable law.

The results of the study may be published in scientific journals or general publications. However, the information concerning your participation will be kept confidential.

**PARTICIPANT'S DECLARATION**. I have been informed by the staff involved in the above-mentioned project:

- Of the advantages and disadvantages, of the purpose of the study, as well as that my results will be provided anonymously to the researchers of the project.

- That I have understood the information I have received and have been able to ask any questions I have deemed appropriate.

**You have the right to participate or not in the research and to withdraw your consent at any time.** I HAVE BEEN PROVIDED WITH A COPY OF THIS DOCUMENT.

Name: ..............................................................................

Signature: Carlos Fernández Morales

# REFERENCES

1. Borghouts JA, Koes BW, Vondeling H, Bouter LM. Cost-of-illness of neck pain in The Netherlands in 1996. Pain. 1999;80(3):629-36.

2. Damgaard P, Bartels EM, Ris I, Christensen R, Juul-Kristensen B. Evidence of Physiotherapy Interventions for Patients with Chronic Neck Pain: A Systematic Review of Randomised Controlled Trials. International Scholarly Research Notices. 15 de abril de 2013;2013:e567175.

3. Espejo-Antúnez L, Fernández-Morales C, Moreno-Vázquez JM, Tabla-Hinojosa FB, Cardero-Durán M de los Á, Albornoz-Cabello M. Assessment from a Biopsychosocial Approach of Flight-Related Neck Pain in Fighter Pilots of Spanish Air Force. An Observational Study. Diagnostics. febrero de 2022;12(2):233.

4. Farrel P, Shender B, Goff C, Baudou J, Crowley J, Davies M. 252 HFaMPNRTG. Aircrew Neck Pain Prevention and Management. NATO Reserach and Technology Organisation. 2019;

5. Van den Oord M. Prevention of flight-related neck pain in military aircrew. 2012.

6. Heneghan NR, Lokhaug SM, Tyros I, Longvastøl S, Rushton A. Clinical reasoning framework for thoracic spine exercise prescription in sport: a systematic review and narrative synthesis. BMJ Open Sport & Exercise Medicine. 1 de marzo de 2020;6(1):e000713.

7. Lange B, Toft P, Myburgh C, Sjøgaard G. Effect of targeted strength, endurance, and coordination exercise on neck and shoulder pain among fighter pilots: a randomized-controlled trial. The Clinical journal of pain. 2013;29(1):50-9.

8. Bahat HS, German D, Palomo G, Gold H, Nir YF. Self-Kinematic Training for Flight-Associated Neck Pain: a Randomized Controlled Trial. Aerospace Medicine and Human Performance. 2020;91(10):790-7.

9. Albornoz-Cabello M, Barrios-Quinta CJ, Espejo-Antúnez L, Escobio-Prieto I, Casuso-Holgado MJ, Heredia-Rizo AM. Immediate clinical benefits of combining therapeutic exercise and interferential therapy in adults with chronic neck pain: a randomized controlled trial. Eur J Phys Rehabil Med. octubre de 2021;57(5):767-74.

10. Blanpied PR, Gross AR, Elliott JM, Devaney LL, Clewley D, Walton DM, et al. Neck pain: revision 2017: clinical practice guidelines linked to the international classification of functioning, disability and health from the orthopaedic section of the American Physical Therapy Association. Journal of Orthopaedic & Sports Physical Therapy. 2017;47(7):A1-83.

11. Lara-Palomo IC, Aguilar-Ferrándiz ME, Matarán-Peñarrocha GA, Saavedra-Hernández M, Granero-Molina J, Fernández-Sola C, et al. Short-term effects of interferential current electro-massage in adults with chronic non-specific low back pain: a randomized controlled trial. Clinical rehabilitation. 2013;27(5):439-49.

12. Espejo-Antúnez L, Fernández-Morales C, Cardero-Durán M de los Á, Toledo-Marhuenda JV, Díaz-Mancha JA, Albornoz-Cabello M. Detection of Changes on Parameters Related to Heart Rate Variability after Applying Current Interferential Therapy in Subjects with Non-Specific Low Back Pain. Diagnostics [Internet]. 2021;11(12). Disponible en: https://www.mdpi.com/2075-4418/11/12/2175

13. Albornoz-Cabello M, Sanchez-Santos JA, Melero-Suarez R, Heredia-Rizo AM, Espejo-Antunez L. Effects of Adding Interferential Therapy Electro-Massage to Usual Care after Surgery in Subacromial Pain Syndrome: A Randomized Clinical Trial. Journal of Clinical Medicine. febrero de 2019;8(2):175.

14. Hussein HM, Alshammari RS, Al-Barak SS, Alshammari ND, Alajlan SN, Althomali OW. A Systematic Review and Meta-analysis Investigating the Pain-Relieving Effect of Interferential Current on Musculoskeletal Pain. Am J Phys Med Rehabil. 2022;101(7):624-633. doi: 10.1097/PHM.0000000000001870.

15. Heng W, Wei F, Liu Z, Yan X, Zhu K, Yang F, et al. Physical exercise improved muscle strength and pain on neck and shoulder in military pilots. Frontiers in Physiology [Internet]. 2022 [citado 13 de diciembre de 2022];13. Disponible en: https://www.frontiersin.org/articles/10.3389/fphys.2022.973304

16. Bradley B, Haladay D. The effects of a laser-guided postural reeducation program on pain, Neck active range of motion, and functional improvement in a 75 year-old patient with cervical dystonia. Physiother Theory Pract 2020;36(4):550-7.

17. Abdollahipour R, Nieto MP, Psotta R, Wulf G. External focus of attention and autonomy support have additive benefits for motor performance in children. Psychology of Sport and Exercise. 2017;32:17-24.

18. Schulz KF, Altman DG, Moher D. CONSORT 2010 statement: updated guidelines for reporting parallel group randomised trials. Journal of Pharmacology and pharmacotherapeutics. 2010;1(2):100-7.

19. Revel M, Andre-Deshays C, Minguet M. Cervicocephalic kinesthetic sensibility in patients with cervical pain. Archives of physical medicine and rehabilitation. 1991;72(5):288-91.

20. Albornoz Cabello M, Rebollo Roldán J, García Pérez R. Escala de Aprensión Psicológica Personal (EAPP) en Fisioterapia. Revista Iberoamericana de Fisioterapia y Kinesiología. 1 de diciembre de 2005;8(2):77-87.

21. Young IA, Cleland JA, Michener LA, Brown C. Reliability, construct validity, and responsiveness of the neck disability index, patient-specific functional scale, and numeric pain rating scale in patients with cervical radiculopathy. American journal of physical medicine & rehabilitation. 2010;89(10):831-9.

22. Andrade Ortega JA, Delgado Martínez AD, Almécija Ruiz R. Validation of the Spanish version of the Neck Disability Index. Spine. 15 de febrero de 2010;35(4):E114-118.

23. Kovacs FM, Bagó J, Royuela A, Seco J, Giménez S, Muriel A, et al. Psychometric characteristics of the Spanish version of instruments to measure neck pain disability. BMC Musculoskelet Disord. 9 de abril de 2008;9:42.

24. Turner JA, Aaron LA. Pain-related catastrophizing: what is it? Clin J Pain. marzo de 2001;17(1):65-71.

25. Tascón M, Estevez R. La interacción entre la distracción y el catastrofismo en la respuesta al dolor. Jiménez A (ed): Comportamiento y palabra, 1a edición Estudios 2005 University, Málaga. 2005;119-21.

26. Gómez-Pérez L, López-Martínez AE, Ruiz-Párraga GT. Psychometric Properties of the Spanish Version of the Tampa Scale for Kinesiophobia (TSK). J Pain. abril de 2011;12(4):425-35.

27. Pousette MW, Lo Martire R, Linder J, Kristoffersson M, Äng BO. Neck muscle strain in air force pilots wearing night vision goggles. Aerospace medicine and human performance. 2016;87(11):928-32.

28. Ang B, Linder J, Harms-Ringdahl K. Neck strength and myoelectric fatigue in fighter and helicopter pilots with a history of neck pain. Aviat Space Environ Med. abril de 2005;76(4):375-80.

29. Molina-Molina A, Ruiz-Malagón EJ, Carrillo-Pérez F, Roche-Seruendo LE, Damas M, Banos O, et al. Validation of mDurance, A Wearable Surface Electromyography System for Muscle Activity Assessment. Frontiers in Physiology. 2020;11:1556.

30. Hermens HJ, Freriks B, Disselhorst-Klug C, Rau G. Development of recommendations for SEMG sensors and sensor placement procedures. Journal of electromyography and Kinesiology. 2000;10(5):361-74.

31. Calamita SAP, Biasotto-Gonzalez DA, De Melo NC, Fumagalli MA, Amorim CF, de Paula Gomes CAF, et al. Immediate effect of acupuncture on electromyographic activity of the upper trapezius muscle and pain in patients with nonspecific neck pain: a randomized, single-blinded, sham-controlled, crossover study. Journal of manipulative and physiological therapeutics. 2018;41(3):208-17.

32. Orellana JN, Torres B de la C, Cachadiña ES, Hoyo M de, Cobo SD. Two New Indexes for the Assessment of Autonomic Balance in Elite Soccer Players. International Journal of Sports Physiology and Performance. 1 de mayo de 2015;10(4):452-7.

33. Torres B de la C, Cabello MA, Bermejo PG, Orellana JN. Autonomic Responses to Ultrasound-Guided Percutaneous Needle Electrolysis of the Patellar Tendon in Healthy Male Footballers: Acupuncture in Medicine [Internet]. 1 de agosto de 2016 [citado 30 de septiembre de 2020]; Disponible en: https://journals.sagepub.com/doi/10.1136/acupmed-2015-010993

34. Espejo-Antúnez L, Fernández-Morales C, Hernández-Sánchez S, Cardero-Durán M de los Á, Toledo-Marhuenda JV, Albornoz-Cabello M. The Impact on the Stress-Associated Autonomic Response of Physiotherapy Students Receiving Interferential Current in an Electrotherapy Training Session. International Journal of Environmental Research and Public Health. enero de 2022;19(20):13348.

35. Font GR, Pedret C, Ramos J, Ortís LC. Variabilidad de la frecuencia cardíaca: concepto, medidas y relación con aspectos clínicos (I). Archivos de medicina del deporte: revista de la Federación Española de Medicina del Deporte y de la Confederación Iberoamericana de Medicina del Deporte. 2008;(123):41-8.

36. Malik M. Heart rate variability: Standards of measurement, physiological interpretation, and clinical use: Task force of the European Society of Cardiology and the North American Society for Pacing and Electrophysiology. Annals of Noninvasive Electrocardiology. 1996;1(2):151-81.

37. Chiviacowsky S, Wulf G, Wally R. An external focus of attention enhances balance learning in older adults. Gait & posture. 2010;32(4):572-5.

38. Slade SC, Dionne CE, Underwood M, Buchbinder R, Beck B, Bennell K, et al. Consensus on exercise reporting template (CERT): modified Delphi study. Physical therapy. 2016;96(10):1514-24.

39. Hoffmann TC, Glasziou PP, Boutron I, Milne R, Perera R, Moher D, et al. Better reporting of interventions: template for intervention description and replication (TIDieR) checklist and guide. Bmj. 2014;348.

40. Modarresi S, Lukacs MJ, Ghodrati M, Salim S, MacDermid JC, Walton DM. A Systematic Review and Synthesis of Psychometric Properties of the Numeric Pain Rating Scale and the Visual Analog Scale for Use in People With Neck Pain. Clin J Pain. 2021 Oct 26;38(2):132-148. doi: 10.1097/AJP.0000000000000999.

41. Cohen J. Statistical power analysis for the behavioral sciences Lawrence Earlbaum Associates. 20th–. Lawrence Earlbaum Associates; 1988.
